# Towards a Structural Basis for the Relationship Between Blood Group and the Severity of El Tor Cholera**

**DOI:** 10.1002/anie.201109068

**Published:** 2012-04-05

**Authors:** Pintu K Mandal, Thomas R Branson, Edward D Hayes, James F Ross, Jose A Gavín, Antonio H Daranas, W Bruce Turnbull

**Affiliations:** Turnbull School of Chemistry and Astbury Centre for Structural Molecular Biology, University of LeedsLeeds, Leeds, LS2 9JT (UK); Instituto Universitario de Bio-Organica “Antonio Gonzalez” Av. AstrofisicoFrancisco Sanchez 2, 38206, La Laguna, Santa Cruz de Tenerife, Canary Islands (Spain)

**Keywords:** bacterial toxin, blood group, calorimetry, carbohydrates

Diarrheal diseases caused by *Vibrio cholerae* and enterotoxigenic *E. coli* (ETEC) lead to millions of deaths each year.^[1]^ The protein toxins produced by these bacteria are 80 % identical and comprise a single toxic A-subunit associated with a pentamer of B-subunits.^[2]^ The B-pentamer enables the toxin to enter cells by first binding to the ganglioside GM1 glycolipid **1** (Figure 1 a,b).^[2]^, ^[3]^ Inhibitors of this binding event are therefore potential anti-diarrheal drugs.^[4]^

**Figure 1:**
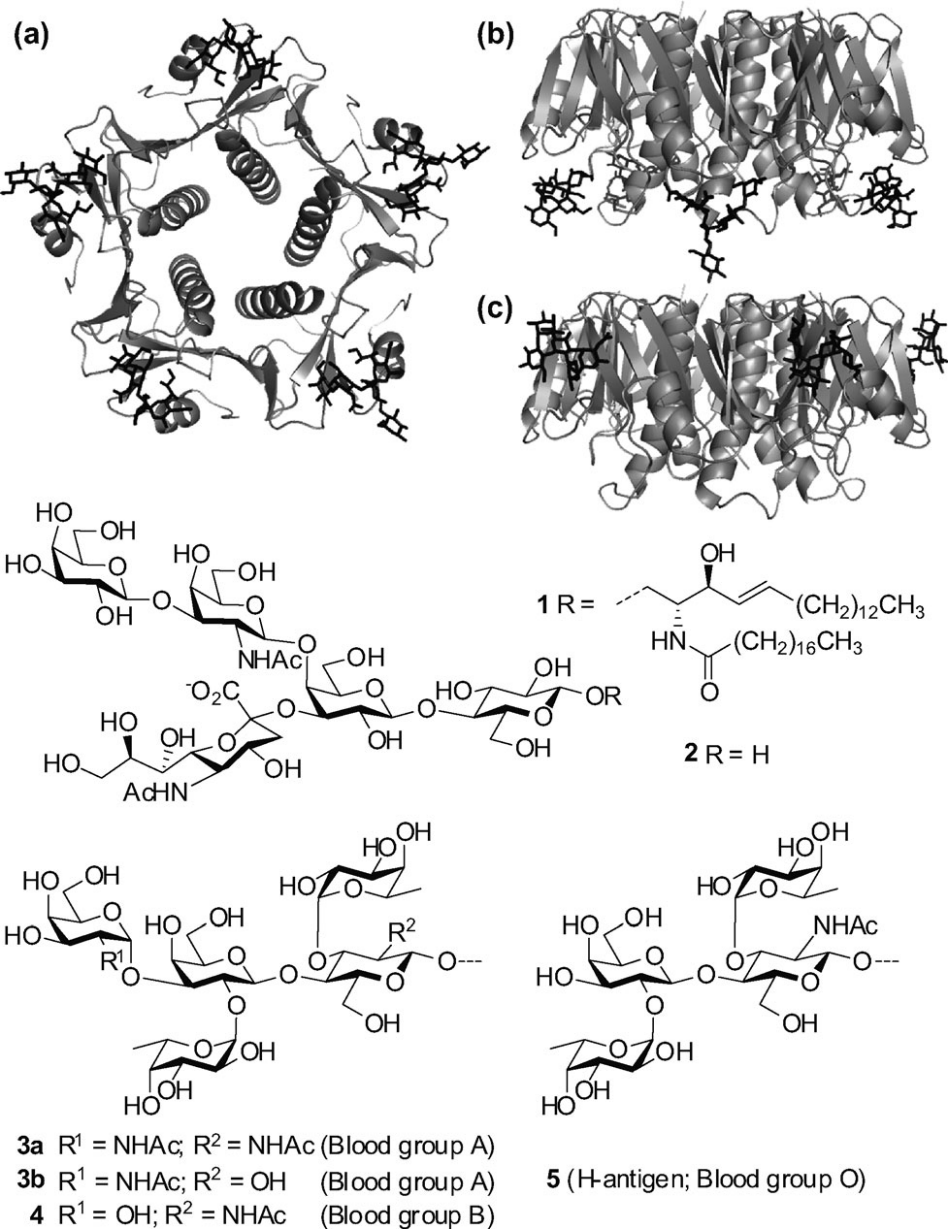
a) Plan and b) elevation views of cholera toxin B-subunit (CTB) with GM1 oligosaccharide 2 bound (3CHB.pdb). c) Elevation view of *E. coli* heat-labile toxin B-subunit (LTB) with blood group A oligosaccharide 3 b analog bound (2O2L.pdb).

The severity of cholera caused by the El Tor biotype of *V. cholerae* is known to be blood-group dependent;^[5]^ people in blood group O are affected more severely than those in blood groups A or B.^[6]^ In contrast, there is no clear blood-group dependence for the *V. cholerae* O1 classical biotype,^[7]^ and any similar correlation for ETEC-related diarrhoea is a matter of dispute.^[8]^ The A, B and O blood groups are distinguished by carbohydrates present on the surface of cells.^[9]^ For example, blood group O is characterized by oligosaccharides terminating in a 2-*O*-fucosyl-galactose structure (e.g. **5**), the so-called H-antigen. In blood groups A and B, the H-antigen is further substituted by an α-galactosamine or galactose residue, respectively (e.g., **3 a** and **4**).

There have been several reports that the cholera toxin B-subunit (CTB) does not bind to blood group oligosaccharides;^[10]^ however, most binding studies appear to have been undertaken using classical biotype CTB, rather than El Tor CTB. In contrast, the heat-labile toxin B-subunit (LTBh) is reported to bind to both blood group A and B oligosaccharides with similar affinity, but not to H-antigen oligosaccharides.^[10a]^,^[b]^ Blood group A oligosaccharide **3 b** has been crystallized with both LTBh and a LTBh/CTB hybrid protein.^[11]^ These studies revealed the presence of a second carbohydrate binding site on the side of the B-pentamers (Figure 1 c).

It has been proposed that the blood-group dependence of El Tor cholera could arise from the toxin being captured by A and B blood group oligosaccharides above the surface of intestinal epithelium cells, thus hindering the toxin from binding to GM1 **1** and entering the cells.^[5]^, ^[11a]^, ^[12]^ We sought to test this hypothesis by measuring the affinities of selected blood group oligosaccharides for El Tor CTB from *V. cholerae* O1/O139^[13]^ and LTBh from *E. coli* H74-114.^[14]^

When studying weakly binding sugars, it is not uncommon for discrepancies to arise between different solid-phase assays as the method of presenting a ligand at a surface can influence its observed affinity for its protein receptor.^[15]^ In such cases, monovalent affinities can often provide more insightful correlations with structural data.^[16]^ Herein we report binding affinities of two monovalent blood group oligosaccharides and propose an alternative explanation for the blood-group dependence of cholera.

Previous studies indicated that both blood group A and B oligosaccharides based on the type-2 Lewis-y core structure were likely to be optimal ligands for LTBh.^[11a]^ As binding had already been confirmed crystallographically for the A oligosaccharide analog **3 a**, we decided to focus our attention on the B pentasaccharide **4**, and Lewis-y tetrasaccharide **5**. Several syntheses of tetrasaccharide **5** have been reported, however, we were surprised to find no published syntheses of pentasaccharide **4** (Scheme 1).

**Scheme 1:**
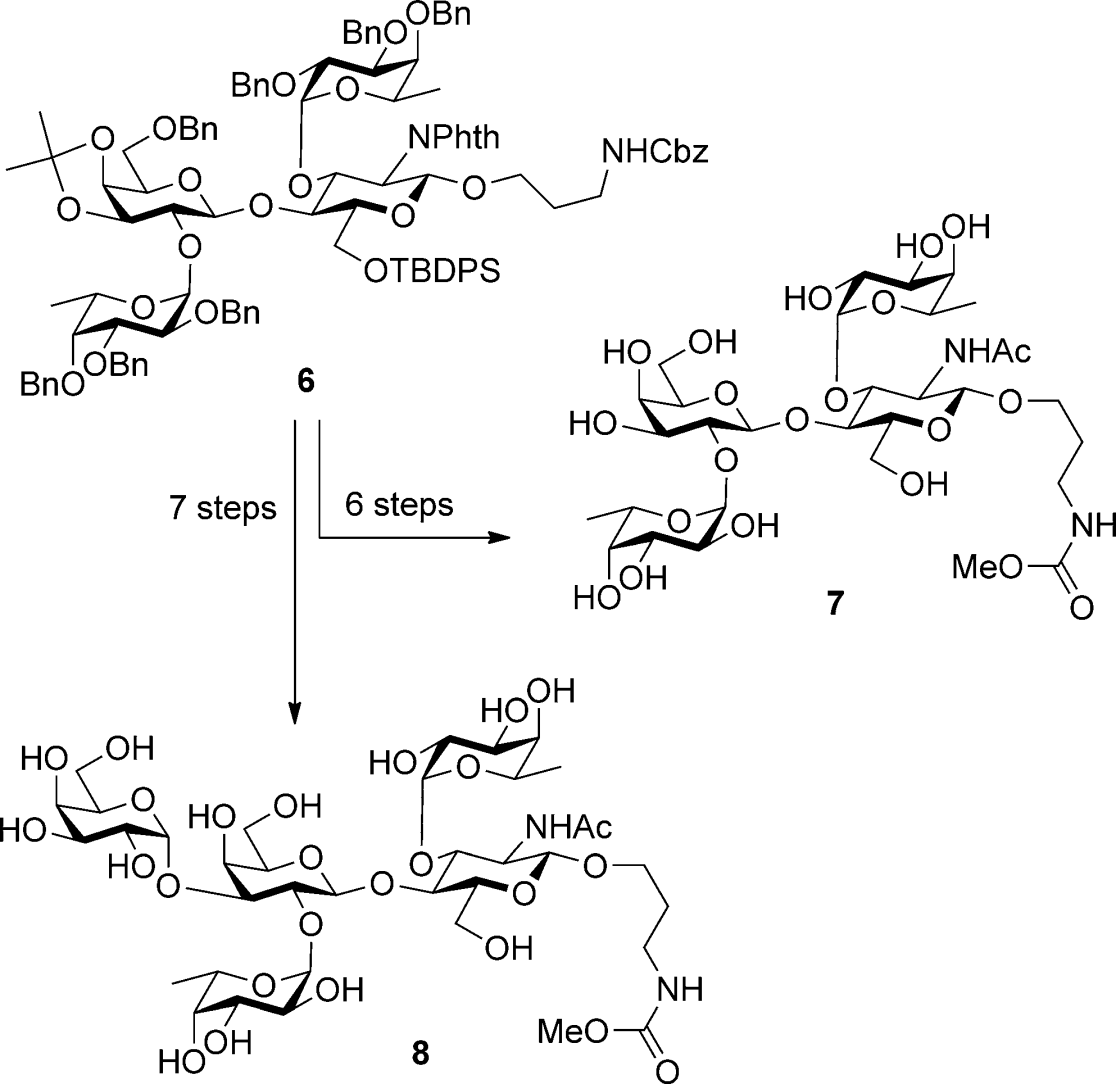
Synthesis of oligosaccharides 7 and 8.

Recently, we developed an efficient synthesis of protected Lewis-y tetrasaccharide **6**,^[18]^ which proved suitable for further elaboration into the target tetrasaccharide **7** and pentasaccharide **8** (see Supporting Information).

Isothermal titration calorimetry (ITC) experiments with the LTBh protein gave hyperbolic curves that are indicative of *K*_d_ being higher than the receptor concentration (Figure 2).^[19]^ The low affinities (Table 1) are perhaps not surprising when one considers that the binding cavity is very shallow. However, even very weak interactions such as these can be functionally relevant when one includes the effect of multivalency. For example, methyl β-galactopyranoside interacts even more weakly with the GM1 binding site (*K*_d_=15 mm),^[16]^ but when displayed on a multivalent scaffold it can inhibit toxin binding with comparable efficiency to the much higher affinity GM1 ligand **2** (monovalent *K*_d_=40 nm).^[20]^ Similarly, the pentameric shiga-like toxin binds its carbohydrate ligand with a 1 mm *K*_d_,^[21]^ yet achieves sub-nanomolar avidity with the corresponding Gb3 glycolipid at a cell surface.

**Figure 2:**
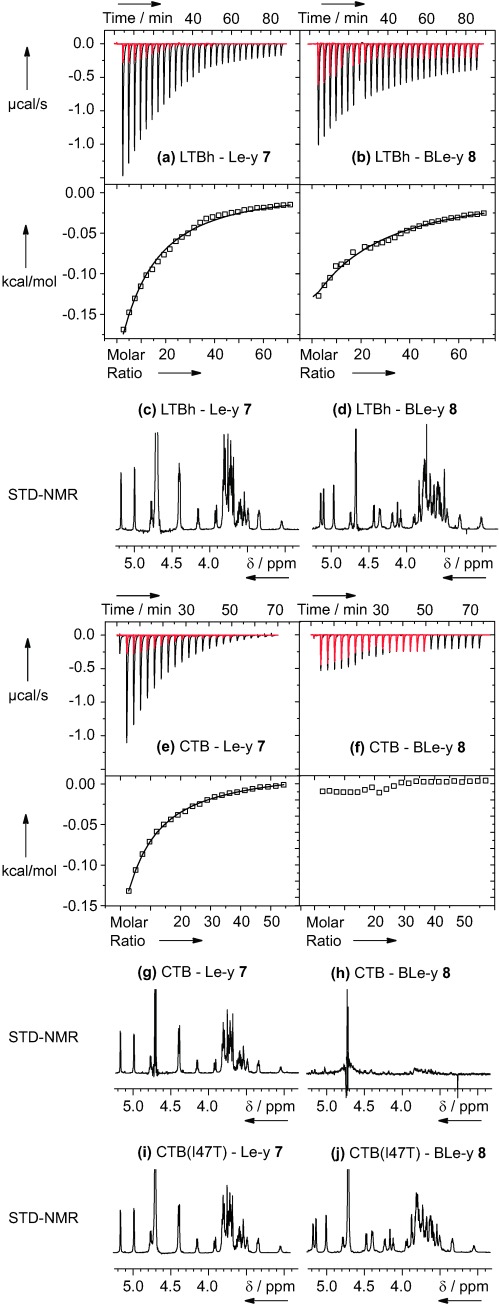
Representative examples of ITC experiments: a) Lewis-y 7 and b) B-Lewis-y 8 titrated into a mixture of LTBh (100 μm) and GM1os (200 μm); c) Lewis-y 7 and d) B-Lewis-y 8 titrated into a solution of El Tor CTB (100 μm). Raw titration data (black) and ligand dilution experiments (red) are shown in the top panels. Bottom panels display the integrated heat data with lines of best fit for each binding curve. c, d, g–j) Saturation transfer difference NMR (STD-NMR) spectra for each titration mixture.

**Table 1:** ITC results for 7 and 8 binding to LTBh and El Tor CTB.

Protein^[a]^	Ligand	+/− **2**^[b]^	*K*_d_ [mm]	Δ*H*° [kcal mol^−1^]
LTBh	**7**	−	7.5±1.9	−5.8±0.5
LTBh	**7**	+	3.0±0.4	−5.9±0.7
El Tor CTB	**7**	−	1.8±0.2	−1.3±0.2
LTBh	**8**	−	5.0±0.5	−8.5±0.9
LTBh	**8**	+	6.7±0.6	−8.8±0.9
El Tor CTB	**8**	−	binding not detected	binding not detected

[a] Titrations were performed at 25 °C in phosphate-buffered saline using 100 μm LTBh/CTB subunit concentration. [b]+ indicates 200 μm GM1os **2** was added to the protein before titration with the ligand.

Contrary to predictions,^[11a]^ tetrasaccharide **7** and pentasaccharide **8** were found to bind to LTBh with comparable affinity. The smaller enthalpy change for **7** is consistent with making fewer interactions with the protein as would be expected for a smaller ligand. To confirm that the ligands were not interacting with the GM1 binding pocket, the titrations were repeated in the presence of saturating concentrations of GM1 oligosaccharide **2**. Only small changes in affinity were observed and the enthalpies of interaction were unchanged. Therefore, ligands **7** and **8** do not interact with the GM1 binding site of LTBh. Instead, they must bind at a different site on the protein which is almost certainly that identified by Krengel and co-workers.^[11a]^ As the GM1 binding pockets on LTBh and El Tor CTB are identical, then any interactions between ligands **7** or **8** and El Tor CTB should also occur at the secondary binding site. It is not clear if the small changes in binding affinity observed when GM1 oligosaccharide **2** is added to the system are really significant. However, it has been reported that a blood group A oligosaccharide and GM1 affect each other’s binding to an unnatural hybrid of LTBh and CTB.^[10c]^

A more surprising result was that tetrasaccharide **7** also binds to El Tor CTB, but pentasaccharide **8** does not (Figure 2 e,f). Whereas ligand **7** gives rise to a saturable binding curve, compound **8** does not show any significant heat of interaction when compared to the control dilution experiment (in gray). Lack of signal in an ITC experiment is not itself proof that a ligand does not bind, as a similar result could be achieved if the ligand were to bind with a very low enthalpy of interaction. Therefore, we sought confirmation of the binding selectivity from a complementary technique. Saturation transfer difference (STD) NMR spectroscopy is widely used to study weakly binding ligands, in particular to determine the orientation in which a protein and ligand interact.^[22]^ The 1D STD NMR spectra confirmed that tetrasaccharide **7** binds to both LTBh and CTB (Figure 2 c,g), whereas pentasaccharide **8** only binds to LTBh (Figure 2 d,h). In addition a quantitative analysis of the STD data indicates that the binding pose of both ligands is similar in both proteins (Supporting Information). In all cases the glucosamine unit shows important and similar STD effects as monitored from protons H1, H5 and the acetamide group whereas H5 and the methyls of both fucose units show poorer STD effects.

The binding results can be rationalized by comparison of the LTBh and CTB structures. All CTB crystal structures in the Protein Data Bank are for classical biotype CTB with a point mutation at position 94 (His94Arg). Therefore models of El Tor CTB were constructed using the crystal structure of either classical biotype CTB (3CHB.pdb)^[23]^ or a CTB–LTBh hybrid protein (3EFX.pdb)^[11b]^ by substitution of appropriate residues in the binding pocket (Figure 3 and Supporting Information). In each case three mutations were made. The models were used purely for a visual comparison with the complex of LTBh and A-Lewis-y **3 b** (2O2L.pdb).^[11a]^ While it is acknowledged that the oligosaccharides could potentially adopt alternative binding poses, in the absence of contrary evidence, it is reasonable to presume that the complexes formed by ligands **7** and **8** should be similar to those in the crystal structure of LTBh and A-Lewis-y **3 b** (2O2L.pdb).^[11a]^

**Figure 3:**
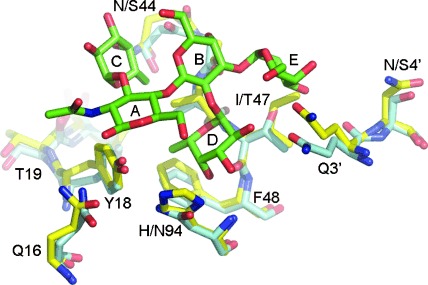
Overlay of LTBh structure (light blue; 2O2L.pdb) and a model of El Tor CTB (yellow) constructed using the structure of classical biotype CTB mutant (3CHB.pdb) by introducing appropriate mutations at positions 18, 47 and 94. The position of oligosaccharide 4 (green) is based on the coordinates of analog 3 b bound to LTBh (2O2L.pdb). Q3′ and N/S4′ are residues in the neighboring B-subunit.

There are four differences in the El Tor CTB and LTBh sequences in the vicinity of the Lewis-y binding pocket. Asparagine-44 (CTB) occupies a similar position to serine-44 (LTBh) and should not interfere with blood group A/B oligosaccharide binding. Indeed, the larger size of N44 (compared to S44) is consistent with the higher STD effect of H1 and H6 of fucose C binding to El Tor CTB. Histidine-94 (CTB) is a relatively conservative change from asparagine-94 (LTBh) and could potentially provide an improved hydrophobic interaction with D-fucose, which also matches well with the higher STD effect of D-fucose in CTB. It is thus not surprising that Lewis-y tetrasaccharide **7** can bind to both proteins. An asparagine at position 4 of a hybrid CTB/LTB protein is able to interact with residue E through a bridging water molecule, thus enhancing binding of blood group A/B oligosaccharides relative to another mutant having serine-4.^[11b]^, ^[24]^ Asparagine-4 (CTB) would thus be expected to promote the interaction with pentasaccharide **8**; however, no binding between CTB and compound **8** was observed experimentally. Therefore, the structural basis for the binding selectivity shown by El Tor CTB could perhaps be attributed to replacing threonine-47 (LTBh) with isoleucine (El Tor CTB). This mutation would result in the loss of a favorable hydrogen bond from the threonine hydroxy group to the 2-NHAc/OH group of residue E,^[11a]^ while potentially introducing a destabilizing steric clash between the isoleucine ethyl group and the 3-OH group of sugar E.

The El Tor CTB Ile47Thr mutant was prepared to test this hypothesis. STD-NMR analysis demonstrated that the mutant protein could bind to both the Lewis-y tetrasaccharide **7** and also the B-Lewis-y pentasaccharide **8** (Figure 2 i,j). Furthermore the STD-NMR data indicated that each oligosaccharide binds in a similar orientation to the complexes with LTBh. ITC analysis showed that the tetrasaccharide **7** binds to the El Tor CTB Ile47Thr mutant with similar affinity to the wild-type protein (*K*_d_=1.0±0.3 mm; Supporting Information). Attempts to measure the affinity of B-Lewis-y **8** by a direct titration were inconclusive (possibly due to a low enthalpy of interaction), but a competition experiment with Lewis-y **7** indicated that the two ligands can bind competitively in the same site (Supporting Information).

Therefore, we conclude that the blood group dependence of El Tor cholera could in part be attributed to a threonine-47-isoleucine mutation in CTB.^[25b]^ This mutation prevents the toxin from binding to blood group B oligosaccharides, contrary to previous predictions. Enterotoxigenic *E. coli* and the *V. cholerae* O1 classical biotype produce toxins that retain threonine-47^[13]^ and should thus show reduced ability to distinguish between blood groups.

Although the monovalent interaction between El Tor CTB and Lewis-y oligosaccharide **7** is weak, it is similar to the monovalent affinity of shiga-like toxin for its ligand.^[21]^ Therefore, we propose that multivalent presentation of the Lewis-y ligand in the glycocalyx will lead to functionally relevant binding. Concentrating the toxin at the cell surface by this mechanism could act as a prelude to entering the cell through the high affinity interaction with ganglioside GM1 **1**. Of course, proof for such a mechanism would require a detailed clinical study which could take other factors into account such as the secretor status of the patient. Nevertheless, our results further our understanding of the blood-group dependence of infectious disease,^[26]^ while presenting new opportunities for developing anti-diarrheal therapies, or even the use of bacterial toxins to target cancers that over-express Lewis-y oligosaccharides.^[27]^

## Notes

Detailed facts of importance to specialist readers are published as ”Supporting Information”. Such documents are peer-reviewed, but not copy-edited or typeset. They are made available as submitted by the authors.
