# Supplementary material for: Towards a Structural Basis for the Relationship Between Blood Group and the Severity of El Tor Cholera
Source: Angew Chem Int Ed Engl. 2012 Apr 5;51(21):5143–6. doi: 10.1002/anie.201109068 (PMC3505909; doi:10.1002/anie.201109068)
Supplement: Supplementary file 1 [file anie0051-5143-sd1.pdf]

Angewandte Chemie
Eine Zeitschrift der Gesellschaft Deutscher Chemiker

Supporting Information
© Wiley-VCH 2012
69451 Weinheim, Germany

Towards a Structural Basis for the Relationship Between Blood Group and the Severity of El Tor Cholera**

*Pintu K. Mandal, Thomas R. Branson, Edward D. Hayes, James F. Ross, Jose A. Gavín, Antonio H. Daranas, and W. Bruce Turnbull**

anie_201109068_sm_miscellaneous_information.pdf

Contents

Synthesis of oligosaccharides **7** and **8** page 2
Protein sequences page 9
Protein expression page 10
Isothermal titration calorimetry page 10
NMR experiments page 12
Comparison of the crystal structure of LTBh with models of El Tor CTB page 41
NMR spectra for intermediates **S1**-**S3** Page 42
References page 45

Synthesis of oligosaccharides 7 and 8

Recently, we developed an efficient synthesis of protected Lewis-y tetrasaccharide **6**,[1] which proved suitable for further elaboration into the target tetrasaccharide **7** and pentasaccharide **8**. Thus, hydrolysis of the acetonide group in **6** and selective orthoester-mediated acetylation of diol **S1** provided acceptor alcohol **S2**. Galactosylation of **S2** using thioglycoside donor **S4** gave pentasaccharide **S3** in 70% yield. A five-step deprotection sequence allowed conversion of compounds **S2** and **S3** into tetrasaccharide **7** and pentsaccharide **8**, which were isolated in good yield as the methyl carbamates. While the methyl carbamate modifications were unexpected, both NMR spectroscopy and mass spectrometry of the final compounds were consistent with these structures.

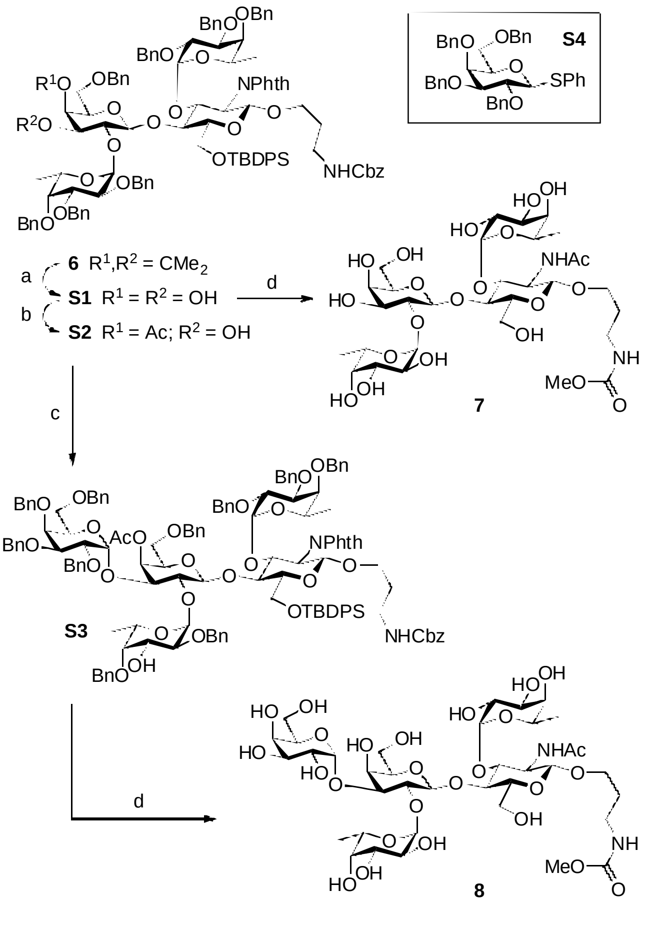

Scheme S1. Reagents and conditions: a) 80% AcOH (aq), 80 °C, 85%; b) (i) $(EtO)_3CH_3$, p-toluenesulfonic acid, DMF, (ii) 80% AcOH (aq), 72% over two steps; c) **6**, NIS, TfOH, MS 4Å, CH_2Cl_2, -40 °C, 70%; d) (i) $H_2NCH_2CH_2NH_2$, n-BuOH, 80 °C, (ii) Ac_2O, C_5H_5N, (iii) NaOMe, MeOH, (iv) TBAF, THF, (v) H_2, 20% $Pd(OH)_2$-C, MeOH; the five step deprotection sequence provided tetrasaccharide **7** in 53%, and pentasaccharide **8** in 50% yield from **S2** and **S3**, respectively.

General methods

All solvents were dried using a PureSolv purification system. All solvents used for flash chromatography were GPR grade, except hexane and ethyl acetate, when HPLC grade was used. All concentrations were performed *in vacuo,* unless otherwise stated. All reactions were performed in oven dried glassware under a N_2(g) atmosphere, unless otherwise stated. Analytical TLC was performed on silica gel 60-F^{254} (Merck) with detection by fluorescence and/or charring following immersion in a 5% H_2SO_4/methanol solution, unless otherwise stated. Optical rotations were measured at the sodium D-line with an Optical Activity AA-1000 polarimeter. $[\alpha]_D$ values are given in units of 10^{-1} deg cm^2 g^{-1}. Infra-red spectra were recorded on a Perkins-Elmer Spectrum One FT-IR spectrometer. Electrospray (ES+) ionisation mass spectra were obtained on a Bruker HCT-Ultra mass spectrometer, and high resolution ES+ were performed on a Bruker Daltonics MicroTOF mass spectrometer.

3-(Benzyloxycarbonylamino)propyl 4-*O*-[6-*O*-benzyl-2-*O*-(2,3,4-tri-*O*-benzyl-α-L-fucopyranosyl)-β-D-galactopyranosyl]-6-*O*-*t*-butyldiphenylsilyl-2-deoxy-2-phthalimido-3-*O*-(2,3,4-tri-*O*-benzyl-α-L-fucopyranosyl)-β-D-glucopyranoside (S1)

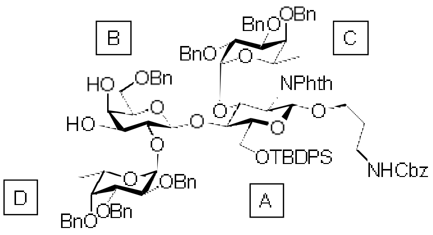

Tetrasaccharide **6**[1] (2.2 g, 1.45 mmol) was dissolved in 80% AcOH (150 mL) and stirred at 80 °C for 2 h. The solution was evaporated to give an oil which was purified over SiO_2 using hexane-EtOAc (2:1) as eluent to furnish diol **S1** as a yellow oil (1.85 g, 85%); $[\alpha]_D^{25}$ -15 (*c* 1.5, $CHCl_3$); IR: 3429, 2931, 1715, 1454, 1386, 1099, 1052, 737, 697 cm^{-1}; ^1H NMR (500 MHz, $CDCl_3$): δ 7.74-7.65 (m, 4 H, Ar-H), 7.41-7.17 (m, 45 H, Ar-H), 7.11 (t, *J* = 7.5 Hz, 1 H, Ar-H), 7.02 (t, *J* = 7.5 Hz, 2 H, Ar-H), 6.93 (d, *J* = 7.5 Hz, 2 H, Ar-H), 5.04-4.99 (m, 3 H, H-1_A, H-1_D, PhCH), 4.96 (d, *J* = 11.6 Hz, 1 H, PhCH), 4.91 (d, *J* = 11.6 Hz, 1 H, PhCH), 4.83-4.72 (m, 8 H, H-1_B, H-1_C, H-5_C, 4 × PhCH), 4.70-4.61 (m, 4 H, H-3_A, 3 × PhCH), 4.58 (d, *J* = 11.6 Hz, 1 H, PhCH), 4.49 (d, *J* = 11.4 Hz, 1 H, PhCH), 4.47-4.41 (m, 4 H, H-2_A, 3 × PhCH), 4.31 (t, *J* = 9.4 Hz, 1 H, H-4_A), 4.23 (d, *J* = 12.2 Hz, 1 H, PhCH), 4.13 (dd, *J* = 10.0, 3.3 Hz, 1H, H-2_D), 4.08-4.06 (br d, *J* = 11.2 Hz, 1 H, H-6_{aA}), 4.01 (dd, *J* = 10.2, 2.6 Hz, H-3_C), 3.89 (br s, 1 H, H-4_B), 3.85-3.69 (m, 7 H, H-6_{bA}, H-6_{aB}, H-6_{bB}, H-2_C, H-4_C, H-3_D, $OC\underline{H}_2CH_2CH_2NH$), 3.51-3.42 (m, 6 H, H-5A, OCH_2, H-2_B, H-3_B, H-5_B, H-4_D, H-2_C, $OC\underline{H}_2CH_2CH_2NH$) 3.31 (q, *J* = 6.4 Hz, 1 H, H-5_D), 3.07-3.05 (m, 2 H, $OCH_2CH_2C\underline{H}_2NH$), 2.14 (s, 1H, OH), 1.70-1.60 (m, 3 H, OH, $OCH_2C\underline{H}_2CH_2NH$), 1.09 (d, *J* = 6.4 Hz, 3 H, 6_C), 1.05 (s, 9 H, $SiC(CH_3)_3$), 0.81 (d, *J* = 6.4 Hz, 3 H, 6_D); ^{13}C NMR (75 MHz, $CDCl_3$): δ 168.6, 168.1, 156.4, 139.0-123.0, 100.8 (2 C), 98.0, 97.5, 81.3, 79.6, 79.1, 78.2, 77.6, 77.3, 75.5, 75.1, 75.0, 74.8, 74.5, 73.7, 73.4, 73.3, 72.9, 72.6, 72.4, 72.2 (2 C), 68.0, 67.9, 67.4, 66.5, 66.4, 66.2, 61.7, 56.5, 38.5, 29.6, 26.9, 19.5, 17.1, 16.9; ESI-MS: $C_{108}H_{118}N_2NaO_{22}Si$ requires *m/z* 1845.7842; found: *m/z* 1845.7855 $[M+Na]^+$.

3-(Benzyloxycarbonylamino)propyl 4-*O*-[4-*O*-acetyl-6-*O*-benzyl-2-*O*-(2,3,4-tri-*O*-benzyl-α-L-fucopyranosyl)-β-D-galactopyranosyl]-6-*O*-*t*-butyldiphenylsilyl-2-deoxy-2-phthalimido-3-*O*-(2,3,4-tri-*O*-benzyl-α-L-fucopyranosyl)-β-D-glucopyranoside (S2)

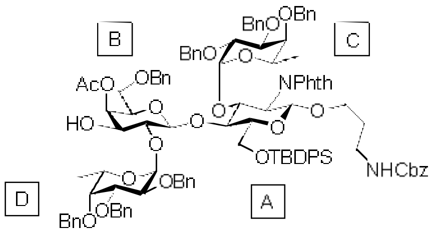

Triethyl orthoacetate (0.3 mL, 1.75 mmol) and p-toluenesulfonic acid (0.3 g, 1.75 mmol) were added to a solution of diol **S1** (1.6 g, 0.877 mmol) in dry DMF (15 mL). The reaction mixture was stirred at room temperature for 2 h. The solvents were removed under reduced pressure and a solution of the intermediate orthoester in 80% aqueous AcOH (50 mL) was stirred at room temperature for 1 h. The reaction mixture was evaporated to dryness and the product was purified over SiO2 using hexane–EtOAc (4:1) as eluant to furnish acetate **S2** as a colourless syrup (1.2 g, 72%); $[\alpha]_D^{25}$ -35 (*c* 1.5, $CHCl_3$); IR: 3428, 2932, 1716, 1651, 1453, 1387, 1098, 750, 698 cm^{-1}; ^{1}H NMR (500 MHz, $CDCl_3$): δ 7.74-7.66 (m, 4 H, Ar-H), 7.34-7.17 (m, 45 H, Ar-H), 7.12 (t, *J* = 7.3 Hz, 1 H, Ar-H), 7.04 (t, *J* = 7.3 Hz, 2 H, Ar-H), 6.94 (m, 2 H, Ar-H), 5.39 (d, *J* = 3.6 Hz, 1 H, H-4_B), 5.05-4.99 (m, 3 H, H-1_A, H-1_D, PhCH), 4.95 (d, *J* = 11.6 Hz, 1 H, PhCH), 4.88-4.86 (m, 2 H, H-1_B, PhCH), 4.81 (d, *J* = 11.7 Hz, 1 H, PhCH), 4.76-4.65 (m, 7 H, H-1_C, H-5_C, H-3_A, 4 × PhCH), 4.61 (d, *J* = 11.6 Hz, 1 H, PhCH), 4.58 (d, *J* = 11.6 Hz, 1 H, PhCH), 4.53 (d, *J* = 11.7 Hz, 1 H, PhCH), 4.48 (d, *J* = 11.4 Hz, 1 H, PhCH), 4.45 (dd, *J* = 8.5, 10.5 Hz, 1 H, H-2_A), 4.40 (brd, *J* = 11.9 Hz, 2 H, 2 × PhCH), 4.33-4.27 (m, 3 H, H-4_A, H-4_D, PhCH), 4.20 (d, *J* = 12.2 Hz, 1 H, PhCH), 4.12-4.09 (m, 2 H, H-6_{aA}, H-2_D), 3.93 (dd, *J* = 2.5, 10.2 Hz, 1 H, H-3_C), 3.87-3.77 (m, 4 H, H-6_{bA}, H-2_C, H-3_D, $OC\underline{H}_2CH_2CH_2NH$), 3.64 (ddd, *J* = 1.6, 3.6, 9.8 Hz, 1 H, H-3_B), 3.62-3.54 (m, 3 H, H-6_{aB}, H-6_{bB}, H-4_C), 3.47-3.39 (m, 4 H, H-5_A, H-2_B, H-5_D, $OC\underline{H}_2CH_2CH_2NH$), 3.32 (t, *J* = 8.4 Hz, 1 H, H-5_B), 3.09-3.05 (m, 2H, $OCH_2CH_2C\underline{H}_2NH$), 1.73-1.58 (m, 5 H, OH, $OCH_2\underline{CH}_2CH_2NH$, $COC\underline{H}_3$), 1.11 (d, *J* = 6.4 Hz, 3 H, H-6_C), 1.06 (s, 9 H, $SiC(CH_3)_3$), 0.89 (d, *J* = 6.4 Hz, 3 H, H-6_D); ^{13}C NMR (75 MHz, $CDCl_3$): δ 169.8, 168.6, 168.1, 156.4, 138.7-123.7, 100.9, 100.6, 98.0, 97.5, 80.8, 79.9, 79.1, 77.7, 77.5, 77.2, 75.5, 74.8, 74.7, 74.6, 74.3, 73.9, 73.4, 72.9 (2 C), 72.8, 72.4, 72.3, 71.7, 69.0, 67.9, 67.2, 66.5 (2 C), 66.2, 61.5, 56.5, 38.4, 29.6, 26.8, 20.8, 19.5, 17.1, 16.8; ESI-MS: $C_{110}H_{120}N_2NaO_{23}Si$ requires *m/z* 1887.7833; found: *m/z* 1887.7846 $[M+Na]^+$.

3-(Benzyloxycarbonylamino)propyl 4-*O*-[4-*O*-acetyl-6-*O*-benzyl-3-*O*-(2,3,4,6-tetra-*O*-benzyl-α-D-galactopyranosyl)-2-*O*-(2,3,4-tri-*O*-benzyl-α-L-fucopyranosyl)-β-D-galactopyranosyl]-6-*O*-*t*-butyldiphenylsilyl-2-deoxy-2-phthalimido-3-*O*-(2,3,4-tri-*O*-benzyl-α-L-fucopyranosyl)-β-D-glucopyranoside (S3)

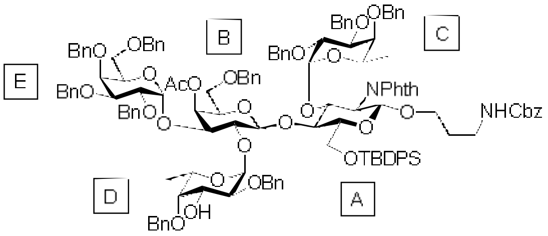

A solution of tetrasaccharide **S2** (1.00 g, 0.54 mmol) and thioglycoside donor **S4** (406 mg, 0.64 mmol) in anhydrous CH_2Cl_2 (30 mL) was strirred with MS-4 Å (500 mg) under N_2 at room temperature for 1 h. The reaction mixture was cooled to −40 °C and N-iodosuccinimide (172 mg, 0.77 mmol) and TfOH (30 μL. 0.18 mmol) were added in succession. The reaction mixture was stirred at same temperature for 1 h before dilution with CH_2Cl_2 (50 mL). The reaction mixture was filtered through a Celite® bed and the organic layer was washed with 5% aq. $Na_2S_2O_3$, satd. $NaHCO_3$ and water, dried (Na_2SO_4) and evaporated to dryness. The crude product was purified over SiO_2 using hexane-EtOAc (6:1) as eluent to furnish pentasaccharide **S3** (900 mg, 70%) as a yellow oil; $[\alpha]_D^{25}$ + 39 (*c* 1.5, $CHCl_3$); IR : 2931, 1715, 1454, 1386, 1238, 1095, 751, 697 cm^{-1}; ^1H NMR (500 MHz, $CDCl_3$): δ 7.69 (d, *J* = 7.0 Hz, 2 H, Ar-H), 7.58 (d, *J* = 7.0 Hz, 2 H, Ar-H), 7.43 (d, *J* = 7.4 Hz, 2 H, Ar-H), 7.33 (d, *J* = 7.4 Hz, 2 H, Ar-H), 7.29-7.02 (m, 50 H, Ar-H), 6.96-6.87 (m, 8 H, Ar-H), 6.82-6.76 (m, 5 H, Ar-H), 6.71-6.67 (m, 4 H, Ar-H), 5.60 (d, *J* = 3.8 Hz, 1 H, H-1_D), 5.58 (d, *J* = 3.0 Hz, 1 H, H-4_B), 5.46 (d, *J* = 3.0 Hz, 1 H, H-1_E), 5.00 (brs, 2 H, 2 × PhCH), 4.93 (d, *J* = 11.2 Hz, 1 H, PhCH), 4.92 (d, *J* = 11.5 Hz, 1 H, PhCH), 4.86 (d, *J* = 7.8 Hz, 1 H, H-1_B), 4.84 (d, *J* = 8.5 Hz, 1 H, H-1_A), 4.80 (d, *J* = 9.1 Hz, 1 H, PhCH), 4.71-4.65 (m, 5 H, H-1_C, H-5_C, 3 × PhCH), 4.63-4.29 (m, 15 H, H-3_A, H-2_A, 13 × PhCH), 4.25-4.11 (m, 6 H, H-4_A, H-6_{aA}, H-5_D, 3 × PhCH), 4.08 (dd, *J* = 2.6, 10.2 Hz, 1 H, H-3_E), 4.04-3.96 (m, 4 H, H-4_E, H-5_E, H-2_D, PhCH), 3.84 (dd, *J* = 2.4, 10.4 Hz, 1 H, H-3_C), 3.81 (dd, *J* = 3.0, 10.2 Hz, 1 H, H-2_E), 3.79-3.71 (m, 4 H, H-2_B, H-3_B, H-6_{aE}, $OC\underline{H_2}CH_2CH_2NH$), 3.69 (dd, *J* = 3.6, 10.4 Hz, 1 H, H-2_C), 3.62 (dd, *J* = 2.5, 10.4 Hz, 1 H, H-3_D), 3.60-3.57 (m, 2 H, H-6_{bA}, H-6_{bE}), 3.48 (br s, 1 H, H-4_C), 3.45-3.34 (m, 4 H, H-6_{aB}, H-6_{bB}, H-4_D, $OC\underline{H_2}CH_2CH_2NH$), 3.29-3.25 (m, 2 H, $OCH_2CH_2C\underline{H_2}NH$), 3.15 (dd, *J* = 7.8, 9.3 Hz, 1 H, H-5_B), 3.05 (br d, *J* = 9.7, 1 H, H-5_A), 1.77-1.70 (m, 2 H, $OCH_2C\underline{H_2}CH_2NH$), 1.67 (s, 3 H, $COC\underline{H_3}$), 1.22 (d, *J* = 6.3 Hz, 3 H, H-6_D), 1.15 (d, *J* = 6.3 Hz, 3 H, H-6_C), 0.81 (s, 9 H, $SiC(CH_3)_3$); ^{13}C NMR (75 MHz, $CDCl_3$): δ 169.5,

168.8, 168.3, 157.5, 138.7-123.7 (Ar-C), 100.3, 98.5, 98.3 (2 C), 97,9, 80.1, 79.4 (2 C), 78.6, 78.2, 77.6, 77.3, 76.8, 76.1, 75.9, 75.7, 75.3, 75.0 (2 C), 74.2 (2 C), 73.9, 73.8 (2 C), 73.4, 73.3 (2 C), 73.2, 73.0, 72.8, 72.7, 71.5, 70.5, 69.5, 68.6, 68.5, 67.5, 67.1, 66.6, 65.7, 61.6, 56.7, 38.5, 29.6, 26.7, 21.2,19.4, 17.0, 16.9; ESI-MS: *m/z* 2411.7 $[M+Na]^+$.

3-(Methyloxycarbonylamino)propyl α-L-fucopyranosyl-(1→2)-β-D-galactopyranosyl-(1→4)-[α-L-fucopyranosyl-(1→3)]-2-acetamido-2-deoxy-β-D-glucopyranoside (7)

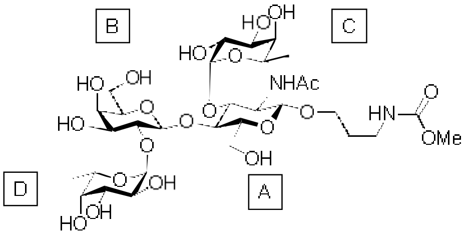

A solution of tetrasaccharide **S1** (450 mg, 0.25 mmol) and ethylene diamine (0.2 mL) in n-butanol (5 mL) was heated to 90 °C for 8 h. The solvents were removed under reduced pressure and a solution of the crude product was re-dissolved in acetic anhydride-pyridine (2 mL; 1:1 v/v) and kept at room temperature for 3 h. The reaction mixture was evaporated and co-evaporated with toluene before treatment with 0.1 M NaOMe in MeOH (5 mL) for 3 h at room temperature. The solution was neutralized using Dowex 50W X-8 (H+) resin, filtered, and concentrated under reduced pressure. The crude mixture was redissolved in tetrabutylammonium fluoride in THF (1 M, 3 mL) and the reaction mixture was stirred at rt for 6 h. The crude product was purified over SiO_2 using hexane–EtOAc (3:1) as eluant. Finally, a solution of the product in CH_3OH (2 mL) was added 20% $Pd(OH)_2$–C (150 mg) and the reaction mixture was allowed to stir at room temperature under a positive pressure of hydrogen for 7 h. The mixture was filtered through a Celite bed and evaporated to dryness before final purification on a Sephadex LH-20 column using CH_3OH–H_2O (8:1 v/v) as eluant to give tetrasaccharide **7** (95 mg, 53%) as a glassy solid; $[\alpha]_D^{25}$ +9.2 (*c* 1.0,D2O); IR: 3440, 2926, 2372, 1429, 1377, 1030, 679 cm^{-1}; ESI-MS: $C_{31}H_{54}N_2NaO_{21}$ requires *m/z* 813.3214; found: *m/z* 813.3221 $[M+Na]^+$. See Table S1 for NMR data.

3-(Methyloxycarbonylamino)propyl α-D-galactopyranosyl-(1→3)-[α-L-fucopyranosyl-(1→2)]-β-D-galactopyranosyl-(1→4)-[α-L-fucopyranosyl-(1→3)]-2-acetamido-2-deoxy-β-D-glucopyranoside (8)

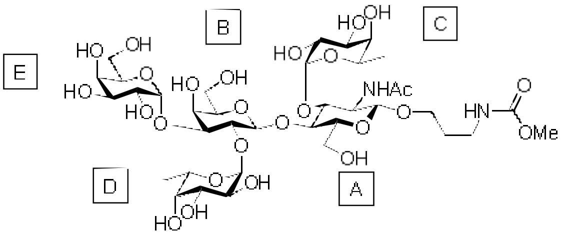

A solution of pentasaccharide **S3** (800 mg, 0.33 mmol) and ethylene diamine (0.2 mL) in n-butanol (10 mL) was heated to 90 °C for 8 h. The solvents were removed under reduced pressure and a solution of the crude product was re-dissolved in acetic anhydride-pyridine (2 mL; 1:1 v/v) and kept at room temperature for 3 h. The reaction mixture was evaporated and co-evaporated with toluene before treatment with 0.1 M NaOMe in MeOH (5 mL) for 3 h at room temperature. The solution was neutralized using Dowex 50W X-8 (H+) resin, filtered, and concentrated under reduced pressure. The crude mixture was redissolved in tetrabutylammonium fluoride in THF (1 M, 4 mL) and the reaction mixture was stirred at rt for 6 h. The crude product was purified over SiO_2 using hexane–EtOAc (4:1) as eluant. Finally, a solution of the product in CH_3OH (2 mL) was added 20% $Pd(OH)_2$–C (150 mg) and the reaction mixture was allowed to stir at room temperature under a positive pressure of hydrogen for 7 h. The mixture was filtered through a Celite bed and evaporated to dryness before final purification on a Sephadex LH-20 column using CH_3OH–H_2O (8:1 v/v) as eluant to give pentasaccharide **8** (150 mg, 50%) as a glassy solid; $[\alpha]_D$ = +32.7 (c 1.0, D_2O); IR: 3446, 2916, 2382, 1419, 1387, 1060, 679 cm^{-1}; ESI-MS: $C_{36}H_{64}N_2NaO_{26}$ requires *m/z* 975.3739; found: *m/z* 975.3751 $[M+Na]^+$. See Table S1 for NMR data.

Protein sequences: LTBh and CTB were expressed from *Vibrio sp.60* clones harbouring plasmids PMMB68[2] and pATA13,[3] respectively, that were kindly provided by Professor T. R. Hirst, Australian National University. The primary structure of each protein was confirmed by subjecting the plasmids to DNA sequencing. The LTBh gene is that derived from an *E. coli* H74-114 isolate of human origin.[2] Its sequence is identical to that found in the 2O2L.pdb crystal structure published by Krengel and co-workers apart from the final residue (Asn103Lys).[4] The CTB gene is identical to that for the toxin from *V. cholerae* O1 El Tor biotype and O139 serogroup.[5] This sequence differs from the toxin derived from the *V. cholerae* O1 classical biotype, at two residues (Tyr18His and Ile47Thr).[5a] The 3CHB.pdb CTB crystal structure published by Hol and co-workers depicts CTB typical of the classical biotype,[6] but with one additional mutation (Arg94His). The I47T mutant was prepared by Quickchange mutagenesis using a plasmid harbouring the El Tor CTB gene. The I47T mutation was confirmed by gene sequencing and also electrospray mass spectrometry of the expressed protein. The amino acid sequences for the mature 103-residue proteins are shown in Figure S1.

```
                  01          11          21          31
LTBh (H74-114)    APQSITELCS  EYHNTQIYTI  NDKILSYTES  MAGKREMVII
LTBh (2O2L.pdb)   APQSITELCS  EYHNTQIYTI  NDKILSYTES  MAGKREMVII
CTB (classical)   TPQNITDLCA  EYHNTQIHTL  NDKIFSYTES  LAGKREMAII
CTB (3CHB.pdb)    TPQNITDLCA  EYHNTQIHTL  NDKIFSYTES  LAGKREMAII
CTB (El Tor)      TPQNITDLCA  EYHNTQIYTL  NDKIFSYTES  LAGKREMAII
CTB (El Tor I47T) TPQNITDLCA  EYHNTQIYTL  NDKIFSYTES  LAGKREMAII
HYB (3EFX.pdb)    APQNITELCS  EYHNTQIYTI  NDKILSYTES  LAGKREMAII

                  41          51          61          71
LTBh (H74-114)    TFKSGATFQV  EVPGSQHIDS  QKKAIERMKD  TLRITYLTET
LTBh (2O2L.pdb)   TFKSGATFQV  EVPGSQHIDS  QKKAIERMKD  TLRITYLTET
CTB (classical)   TFKNGATFQV  EVPGSQHIDS  QKKAIERMKD  TLRIAYLTEA
CTB (3CHB.pdb)    TFKNGATFQV  EVPGSQHIDS  QKKAIERMKD  TLRIAYLTEA
CTB (El Tor)      TFKNGAIFQV  EVPGSQHIDS  QKKAIERMKD  TLRIAYLTEA
CTB (El Tor I47T) TFKNGATFQV  EVPGSQHIDS  QKKAIERMKD  TLRIAYLTEA
HYB (3EFX.pdb)    TFKNGATFQV  EVPGSQHIDS  QKKAIERMKD  TLRIAYLTEA

                  81          91         101
LTBh (H74-114)    KIDKLCVWNN  KTPNSIAAIS  MEN
LTBh (2O2L.pdb)   KIDKLCVWNN  KTPNSIAAIS  MEK
CTB (classical)   KVEKLCVWNN  KTPHAIAAIS  MAN
CTB (3CHB.pdb)    KVEKLCVWNN  KTPRAIAAIS  MAN
CTB (El Tor)      KVEKLCVWNN  KTPHAIAAIS  MAN
CTB (El Tor I47T) KVEKLCVWNN  KTPHAIAAIS  MAN
HYB (3EFX.pdb)    KVEKLCVWNN  KTPNSIAAIS  MAN
```

Figure S1. Amino acid sequences of LTBh and CTB variants. LTBh (H74-114) and CTB (El Tor) were used in the current study. Differences between the CTB sequences are highlighted in green and differences between the LTBh sequences are highlighted in blue.

Protein expression: A single bacterial colony of the appropriate clone was used to inoculate a flask of high salt LB growth media (100 mL). The starter culture was incubated at 30 °C for 20 h before an aliquot (20 mL) was added to 10 x 1 L growth media. The cultures were then incubated at 30 °C until the OD_{600} had reached ~0.5, at which time IPTG (120 mg) was added to each flask to induce protein over-expression. After incubating for a further 20 h, the cells were isolated by centrifugation at 38,000 x g for 20 min. The bacterial pellet was discarded and sodium hexametaphosphate (2.5 g L^{-1}) was added to the supernatant and the pH was adjusted to 3.5 by addition of HCl. The solution was then stored at 4 °C for 4 days to allow the protein to precipitate. Alternatively, the protein was recovered by ammonium sulfate precipitation. The suspension was centrifuged at 16,000 x g for 45 min, before discarding the supernatant and the protein pellet was resuspended in TRIS buffer (20 mL). This solution was centrifuged at 110,500 x g for 30 min. The supernatant was collected and filtered through a 0.45 µm filter (Sartorius Minisart). The protein solution was purified on a lactose affinity column (Sigma) and eluted with TEAN buffer (pH 7.0: 50 mM Tris, 200 mM NaCl, 3 mM NaN_3, 1 mM EDTA) containing 300 mM lactose. The purity of the protein was determined to be >95% by SDS-PAGE.

Isothermal titration calorimetry: Protein solutions were dialysed into phosphate buffered saline (pH 7.4) using SnakeSkin® pleated dialysis tubing (Thermo Scientific) with 7000 MWCO. The buffer solution was changed three times to ensure effective dialysis. ITC experiments were performed using either a VP-ITC or a iTC200calorimeter (GE Healthcare), with a cell volume of 1.409 mL and 0.2028 mL, respectively. All titrations were conducted at 25 ºC using a CTB or LTBh subunit concentration of 100 μM (i.e. 20 μM pentamer concentration). Ligands **7** and **8** were dissolved in the buffer from the final dialysis step. Titrations, typically run in duplícate, comprised 25-30 injections of 8 μL at 4 minute (or 2 μL at 2 minute) intervals. Separate titrations of ligands into the same buffer were used to subtract the heat of dilution from the integrated data prior to curve fitting in Origin using the one site model. The binding stoichiometry (n) was fixed at 1.0 for low c-value titrations as described by Turnbull and Daranas.[7]

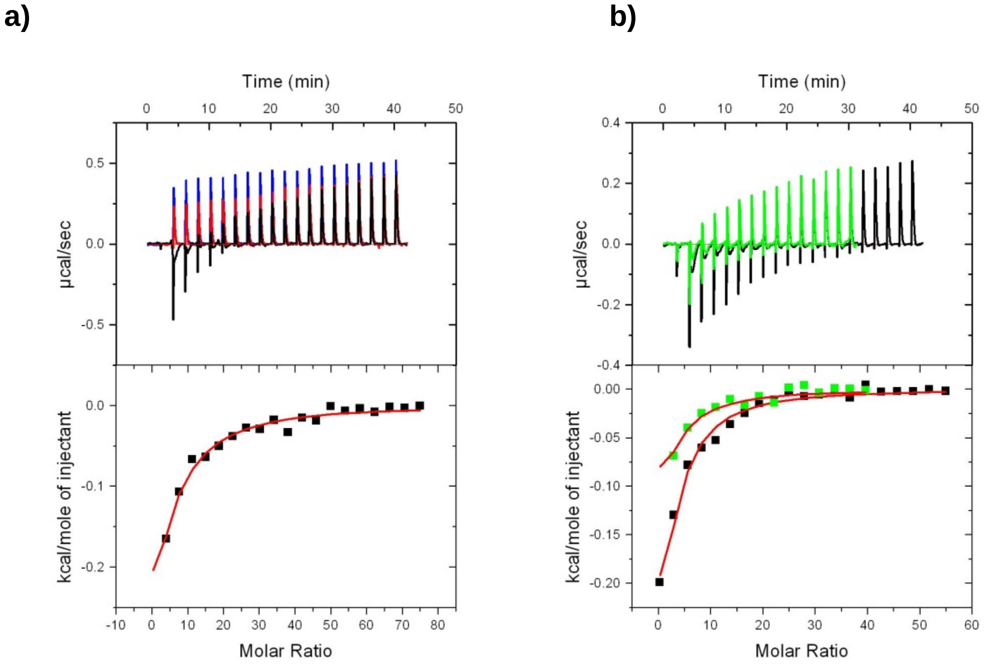

Figure S2. ITC titrations using the Ile47Thr El Tor CTB mutant. (a) titration of 32 mM tetrasaccharide **7** into 120 μM B-subunit (black) with corresponding dilution experiment (red) and control titration of 180 mM D_2O into water (blue). (b) Titration of 32 mM tetrasaccharide **7** into 120 μM B-subunit (black) overlaid with a competition titration experiment in which 32 mM tetrasaccharide **7** was titrated into a mixture of 120 μM B-subunit and 5.5 mM pentasaccharide **8**.

Figure S2 shows titrations using the Ile47Thr El Tor CTB mutant protein. The positive heat of dilution in panel (a) arises from a slight buffer mismatch that was a consequence of freeze-drying the oligosaccharide from a D_2O NMR sample which leads to the hydroxyl group protons exchanging for deuterium. The control titration curve shown in blue represents the amount of heat that is released when a corresponding concentration of D_2O is diluted in water. This minor buffer mismatch has no impact on the processed ITC data (Fig. S2a lower panel).

Tetrasaccharide **7** was found to bind with K_d = 1.0 ±0.3 mM, ΔH = 2.3 ±0.8 kcal/mol.

When tetrasaccharide **7** is titrated into a mixture of pentasaccharide **8** and the mutant protein the enthalpy changes decrease (green line Fig S2b) relative to a titration in the absence of pentasaccharide **8** (black line Fig S2b). This result is consistent with the STD-NMR experiments which suggest that both oligosaccharides can bind in the same site on the protein. However, the small difference in the ITC curves (Fig S2) was insufficient to allow quantitative determination of the affinity and enthalpy change for binding of the pentasaccharide.

NMR sample preparation: Protected carbohydrate intermediates were dissolved in deuterated chloroform. Final unprotected carbohydrates were dissolved in D_2O (99.97%, Eurisotop, France). Protein-ligand complexes were dissolved in deuterated phosphate buffered saline (PBS) at pH 7.4 to give a protein concentration of 60 μM (or 30 μM for the I47T mutant). A 500:1 excess ratio of ligand was used.

NMR data acquisition and analysis: ^1H NMR spectra for protected carbohydrates were acquired at 500 MHz on a Bruker Avance 500 instrument or at 300 MHz on a Bruker Avance 300 instrument. ^{13}C NMR spectra for protected carbohydrates were acquired at 75 MHz on a Bruker Avance 300 instrument. Chemical shifts are given in parts per million downfield from tetramethylsilane. Monosaccharide residues are labelled A-E as indicated in the structures accompanying each synthetic procedure. All NMR measurements on final unprotected carbohydrates were performed on a Bruker AVANCE III 600 MHz spectrometer equipped with a 5 mm TCI cryogenic probe using 3 mm NMR tubes. The temperature was set at 300 K for all experiments. Topspin 3.0 was used for data acquisition, processing and analysis. Standard pulse sequences from the Bruker library were used. All experiments were acquired with 2048 points in the F2 dimension and 256 to 512 increments in the F1 dimension. HMBC was optimized for a long range coupling constant of 7 Hz. TOCSY mixing time was set to 60 ms with an excitation bandwith of 8000 Hz. Zero Quantum filtered 1D-TOCSY were acquired using Gaussian selective pulses and a mixing time of 80 ms. A mixing time of 300 ms was used in the NOESY experiment.

All STD experiments, were acquired using a train of Gaussian shaped pulses of 48 ms, spaced by 1.0 ms delays, with an excitation bandwidth of 55 Hz. Saturation times of 5.0 s and a 10 ms $T1\ \rho$ filter ($\gamma B_1/2\pi$ = 6000 Hz) was used to remove residual protein signals. On-resonance irradiation was performed both at -1 ppm and 7.5 ppm and off-resonance irradiation was set at 30.0 ppm. Appropriate blank experiments were also performed to assure the absence of direct irradiation on the ligand. Analysis of the STD data was done using the CLEAN-STD approach in order to avoid power spillover in those halves of the spectra closer to the on-resonance RF irradiation frequency.

Table S1. Chemical shifts of Lewis-y (**7**) and BLewis-y (**8**) in D_2O.

	Lewis-y **7**				BLewis-y **8**		
Position	δ_H	δ_C	Multiplicity. *J* (Hz)	Position	δ_H	δ_C	Multiplicity. *J* (Hz)
H1-GlcNAc-A	4.39	100.9	d, 8.0	H1-GlcNAc-A	4.39	100.8	d, 8.0
H2-GlcNAc-A	3.77	55.9		H2-GlcNAc-A	3.76	56.0	
H3-GlcNAc-A	3.48	74.8		H3-GlcNAc-A	3.63	74.8	
H4-GlcNAc-A	3.80	73.3		H4-GlcNAc-A	3.80	73.9	
H5-GlcNAc-A	3.34	75.5		H5-GlcNAc-A	3.33	75.5	
H6-GlcNAc-A	3.72	59.9		H6-GlcNAc-A	3.73	59.9	
H6'-GlcNAc-A	3.91	59.9	dd, 1.6, 12.0	H6'-GlcNAc-A	3.93	59.9	dd, 1.6, 12.0
H1-Gal-B	4.39	100.2	d, 8.0	H1-Gal-B	4.46	100.3	d, 7.6
H2-Gal-B	3.54	76.2		H2-Gal-B	3.77	72.7	
H3-Gal-B	3.74	73.5		H3-Gal-B	3.86	76.1	dd, 2.0, 9.5
H4-Gal-B	3.75	68.7		H4-Gal-B	4.15	63.1	
H5-Gal-B	3.71	74.8		H5-Gal-B	3.50	74.5	
H6-Gal-B	3.60	61.4		H6-Gal-B	3.60	61.3	
H1-Fuc-C	4.99	98.6	d, 3.9	H1-Fuc-C	4.99	98.5	d, 3.7
H2-Fuc-C	3.58	67.6		H2-Fuc-C	3.58	67.6	
H3-Fuc-C	3.80	69.1		H3-Fuc-C	3.80	69.0	
H4-Fuc-C	3.69	71.9		H4-Fuc-C	3.66	72.0	
H5-Fuc-C	4.77	66.8	q, 6.3	H5-Fuc-C	4.77	66.8	q, 6.6
H6-Fuc-C	1.13	15.4	d, 6.3	H6-Fuc-C	1.13	15.5	d, 6.6
H1-Fuc-D	5.16	99.3	d, 3.1	H1-Fuc-D	5.17	98.9	d, 4.1
H2-Fuc-D	3.68	68.2		H2-Fuc-D	3.68	67.7	
H3-Fuc-D	3.68	69.7		H3-Fuc-D	3.60	70.0	
H4-Fuc-D	3.71	71.6		H4-Fuc-D	3.71	71.7	
H5-Fuc-D	4.15	66.8	q, 6.2	H5-Fuc-D	4.22	66.8	dd, 6.1, 6.5
H6-Fuc-D	1.16	15.4	q, 6.2	H6-Fuc-D	1.18	15.3	d, 6.5
O-CH_2	3.49	67.8		H1-Gal-E	5.13	92.9	d, 3.1
	3.81	67.8		H2-Gal-E	3.78	69.4	
CH_2	1.63	28.7	q, 6.5	H3-Gal-E	3.77	68.0	
N-CH_2	3.04	37.3	dddd, 6.7	H4-Gal-E	3.86	69.2	
Acetyl-Me	1.92	22.1	s	H5-Gal-E	4.11	71.0	dd, 6.1.6.1
Acetyl-CO		174.4		H6-Gal-E	3.62	61.1	d, 6.1
Carbamate-OMe	3.53	52.3		O-CH_2	3.50	67.8	
Carbamate-CO		159.2			3.82	67.8	
				CH_2	1.63	28.7	t, 6.5
				N-CH_2	3.04	37.3	dddd, 6.5
				Acetyl-Me	1.92	22.1	s
				Acetyl-CO		174.3	
				Carbamate-OMe	3.54	52.3	
				Carbamate-CO		159.2	

Table S2. Normalized STD amplification factors for the LTBh-BLewis-y. LTBh-Lewis-y and CTB-Lewis-y complexes.

LTBh-BLewis-y		LTBh-Lewis-y		CTB-Lewis-y	
100.0	H5-GlcNAc-A	100.0	H5-GlcNAc-A	100.0	H5-GlcNAc-A
55.9	H6-GlcNAc-A	89.5	H6-GlcNAc-A	70.5	H6-GlcNAc-A
110.1	H1-GlcNAc-A	140.8	H1-GlcNAc-A/ H1-Gal-B	131.2	H1-GlcNAc-A/ H1-Gal-B
63.8	H1-Gal-B				
59.7	H4-Gal-B				
78.3	H1-Fuc-C	87.7	H1-Fuc-C	96.0	H1-Fuc-C
47.9	H5-Fuc-C	62.6	H5-Fuc-C	59.2	H5-Fuc-C
33.1	H6-Fuc-C	31.8	H6-Fuc-C	38.8	H6-Fuc-C
60.2	H1-Fuc-D	58.3	H1-Fuc-D	95.3	H1-Fuc-D
62.3	H5-Fuc-D	64.2	H5-Fuc-D	74.0	H5-Fuc-D
50.1	H6-Fuc-D	32.8	H6-Fuc-D	45.9	H6-Fuc-D
54.8	H1-Gal-E				
52.4	H5-Gal-E				
29.6	CH_2	31.7	CH_2	29.8	CH_2
33.2	N-CH_2	38.1	N-CH_2	39.6	N-CH_2
79.8	Acetyl	61.8	Acetyl	71.7	Acetyl

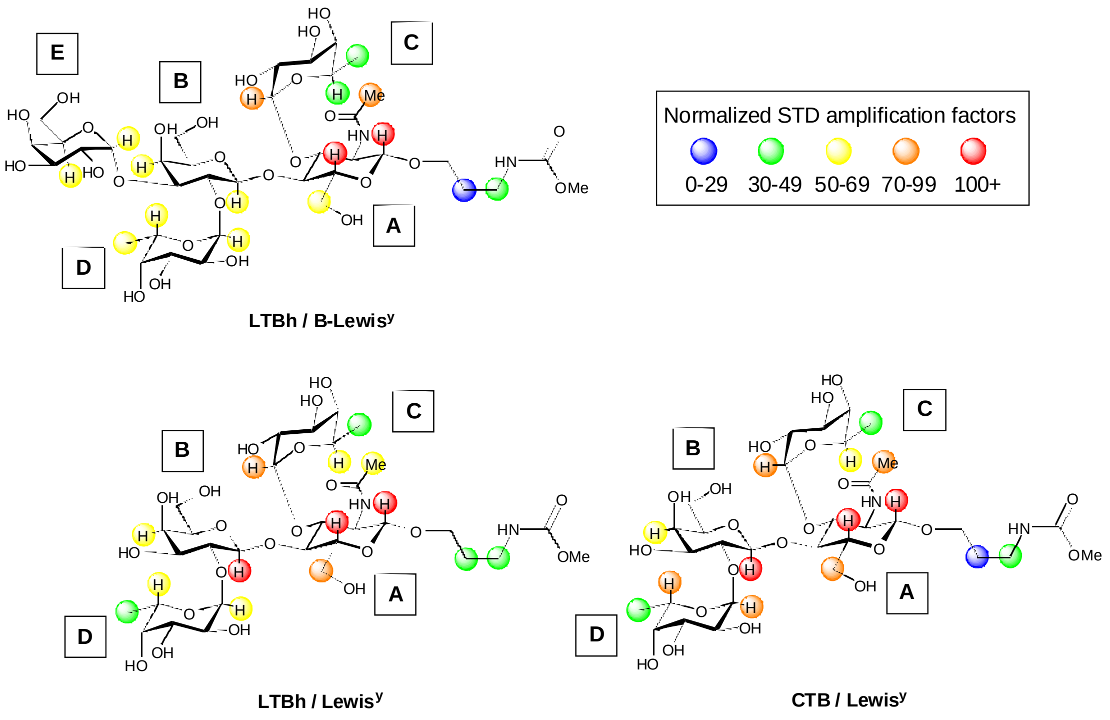

Fig S3. Normalized STD amplification factors for the LTBh-BLewis-y. LTBh-Lewis-y and CTB-Lewis-y complexes.

Table S3. Normalized STD amplification factors for the CTB-I47T-BLewis-y and CTBI47T-Lewis-y complexes.

CTB-I47T-BLewis-y		CTB-I47T -Lewis-y	
100.0	H5-GlcNAc-A	100.0	H5-GlcNAc-A
55.4	H6-GlcNAc-A	74.2	H6-GlcNAc-A
114.2	H1-GlcNAc-A	129.5	H1-GlcNAc-A/ H1-Gal-B
66.9	H1-Gal-B		
57.2	H4-Gal-B		
83.7	H1-Fuc-C	97.2	H1-Fuc-C
60.9	H5-Fuc-C	91.5	H5-Fuc-C
31.6	H6-Fuc-C	26.0	H6-Fuc-C
61.7	H1-Fuc-D	75.1	H1-Fuc-D
62.5	H5-Fuc-D	69.8	H5-Fuc-D
39.8	H6-Fuc-D	25.7	H6-Fuc-D
51.1	H1-Gal-E		
44.7	H5-Gal-E		
23.0	CH_2	20.7	CH_2
27.7	N-CH_2	27.2	N-CH_2
64.8	Acetyl	50.3	Acetyl

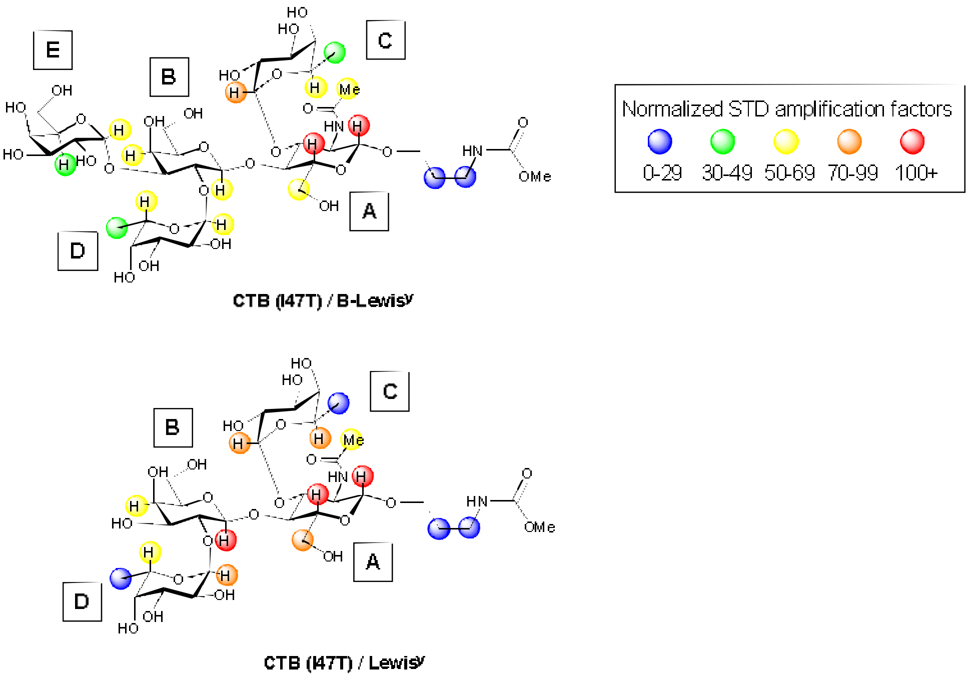

Fig S4. Normalized STD amplification factors for the CTB-I47T-BLewis-y and CTBI47T-Lewis-y complexes.

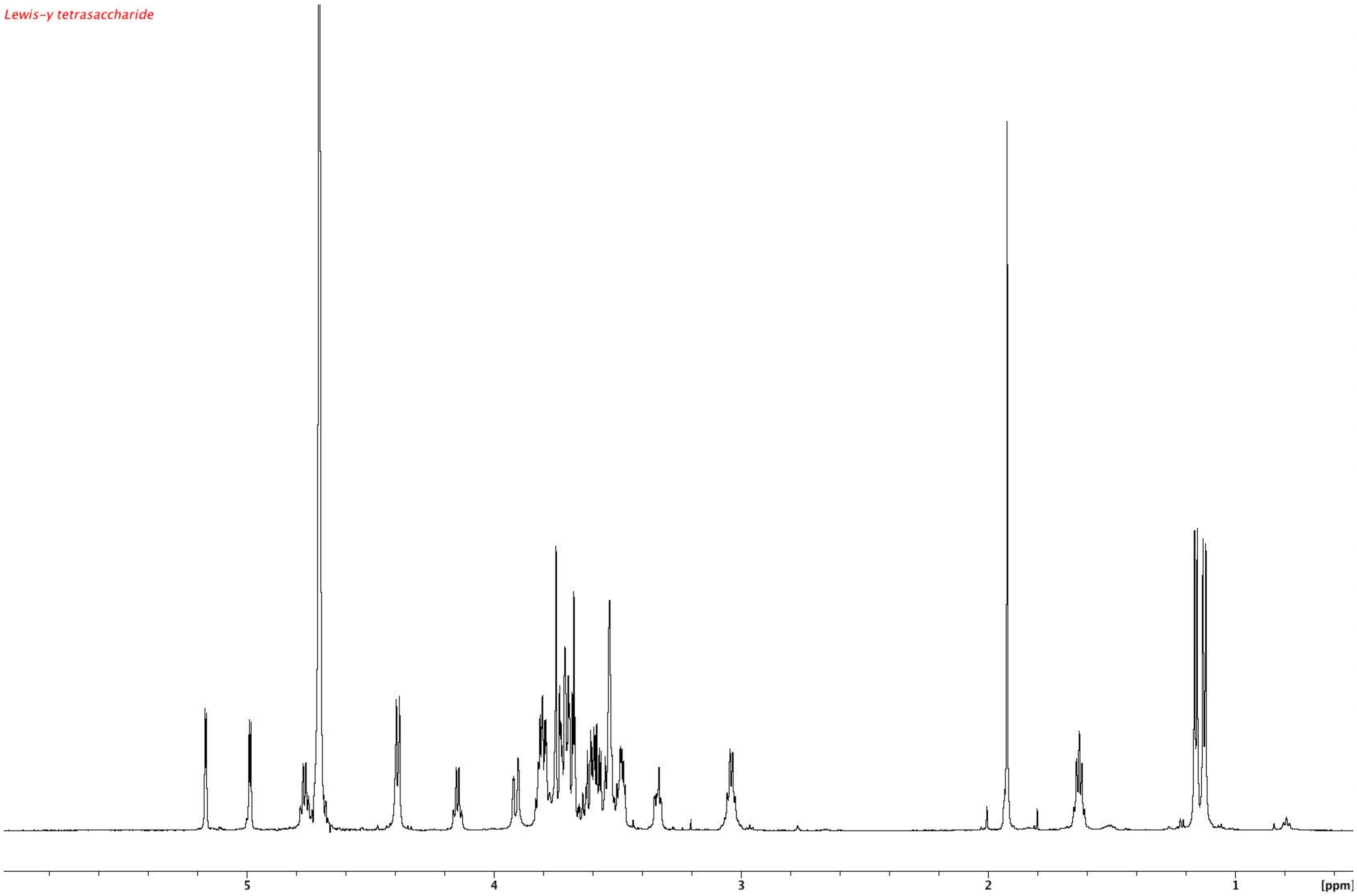

Figure S5: ^{1}H NMR spectrum of Lewis-y tetrasaccharide **7**.

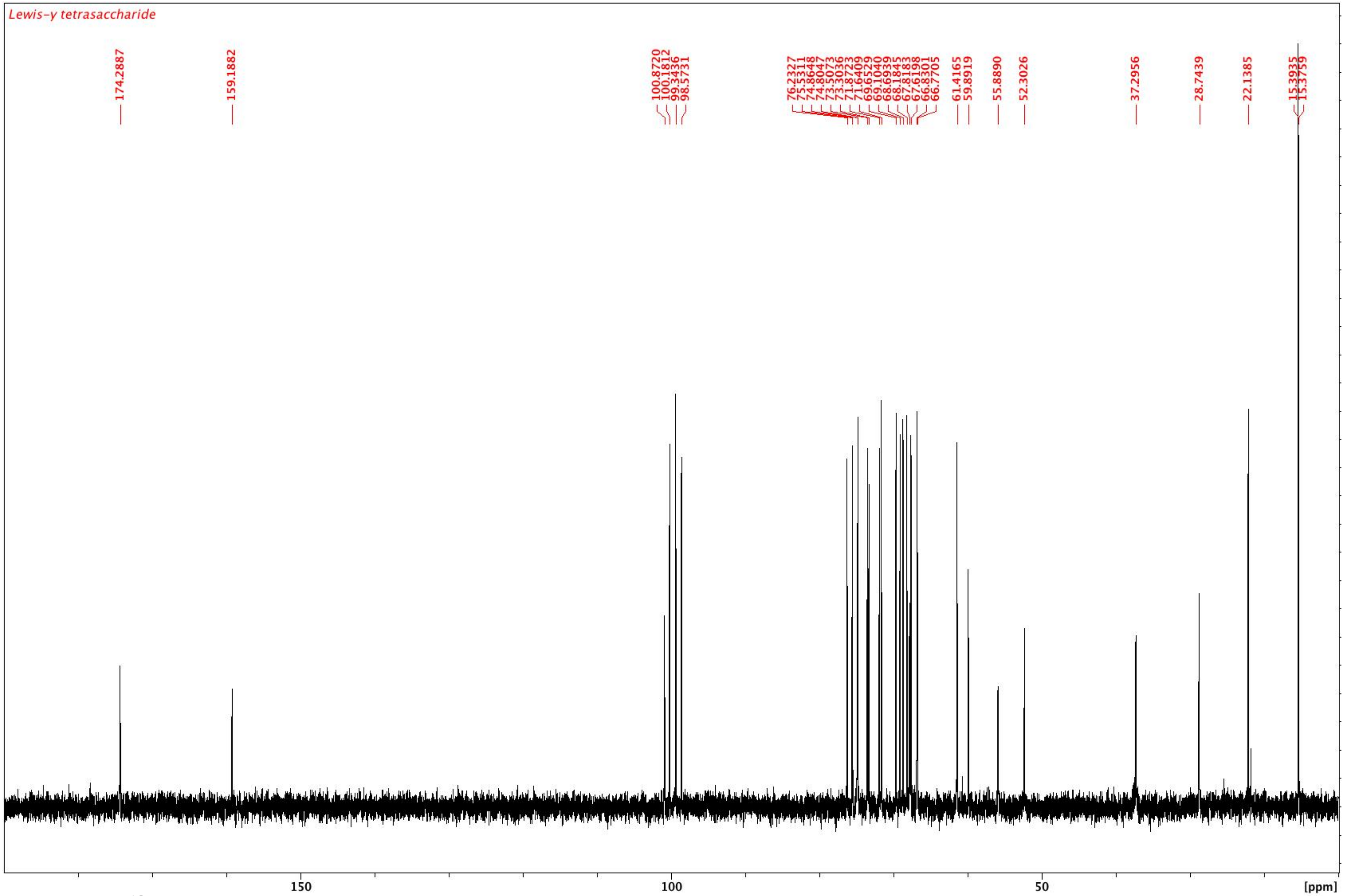

Figure S6: ^{13}C NMR spectrum of Lewis-y tetrasaccharide **7**.

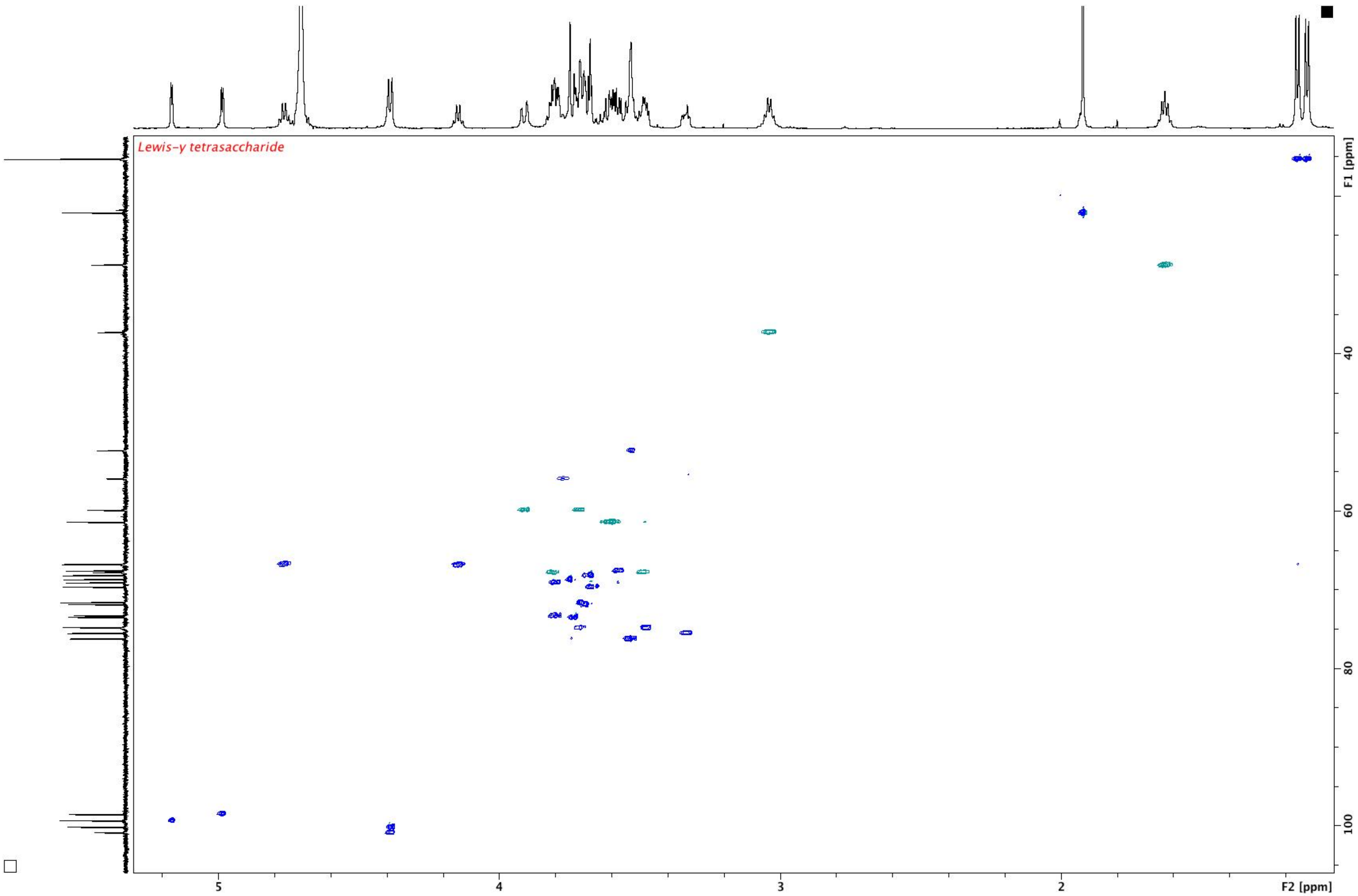

Figure S7: HSQC-edited of Lewis-y tetrasaccharide **7**.

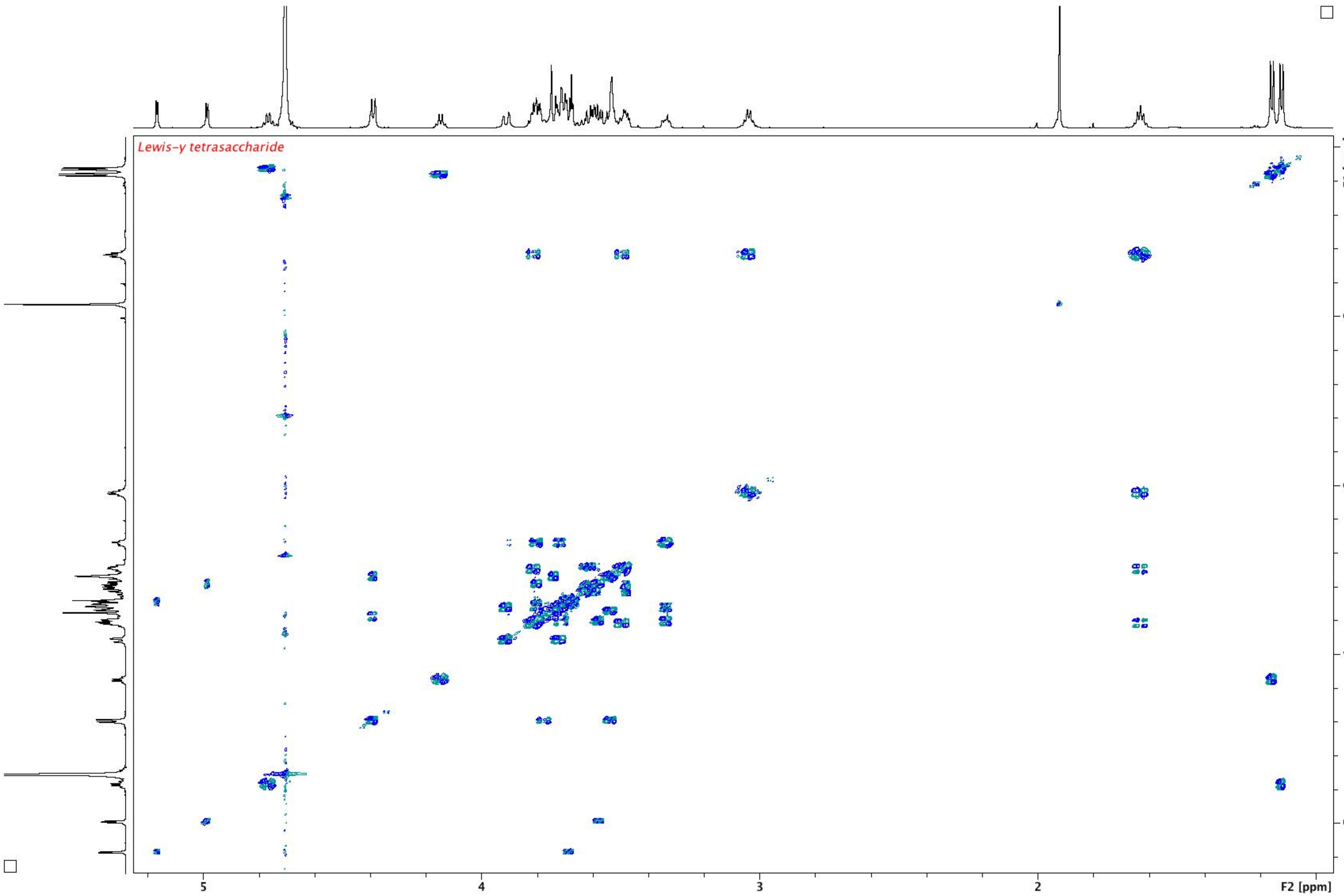

Figure S8: DQF-COSY of Lewis-y tetrasaccharide **7**.

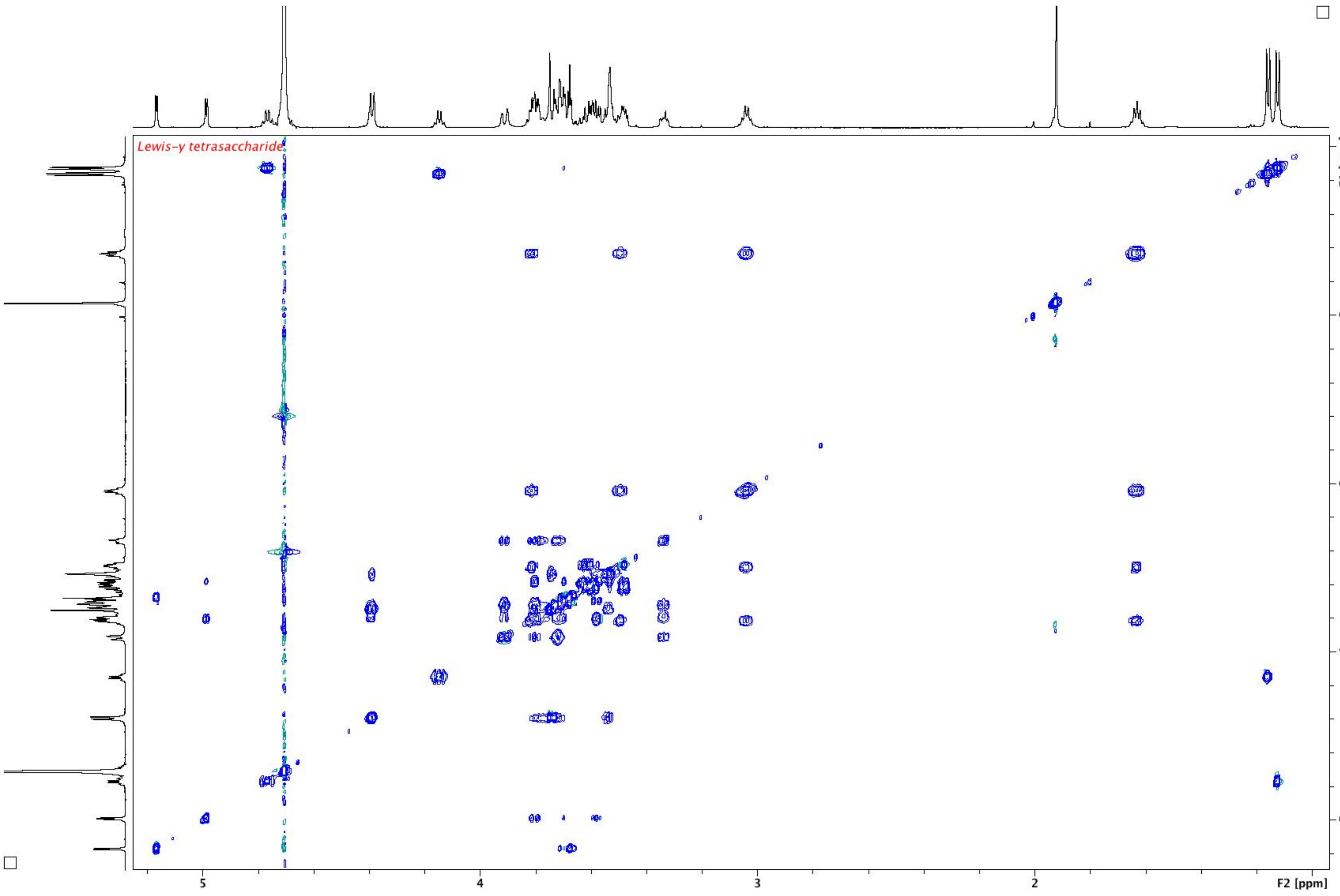

FigureS9: TOCSY of Lewis-y tetrasaccharide **7**.

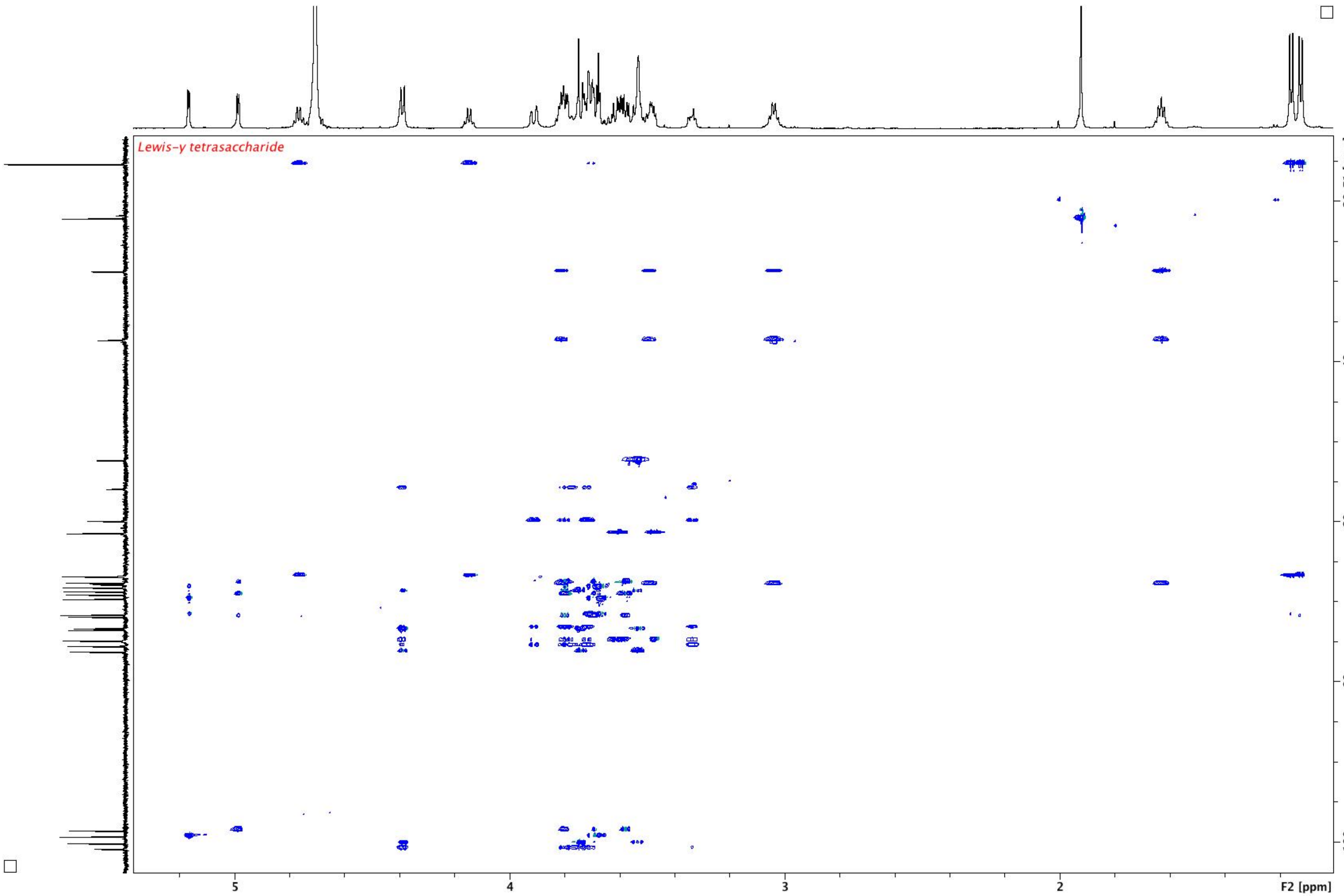

Figure S10: HSQC-TOCSY of Lewis-y tetrasaccharide **7**.

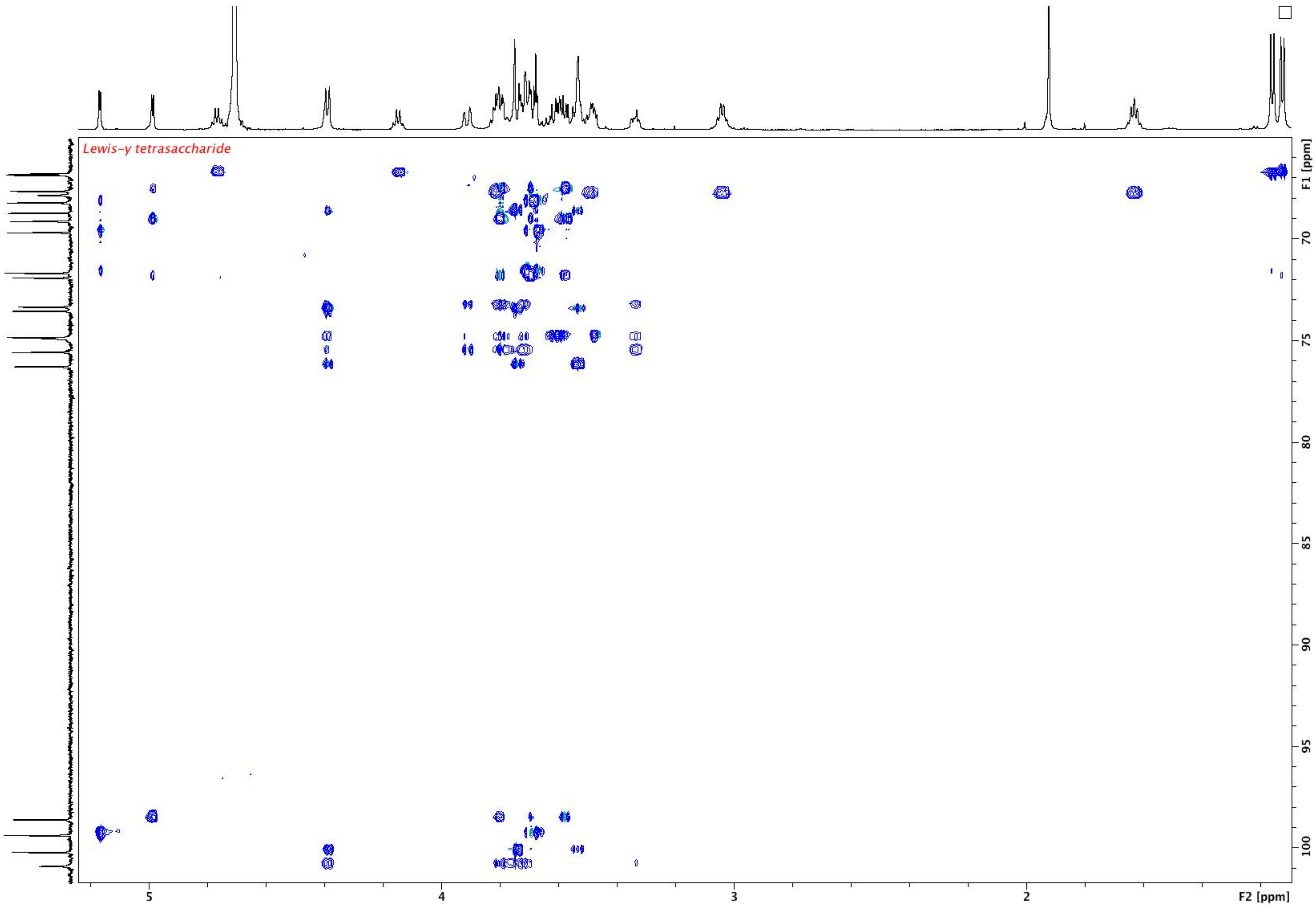

Figure S11: Expansion of the HSQC-TOCSY spectrum of Lewis-y tetrasaccharide **7**.

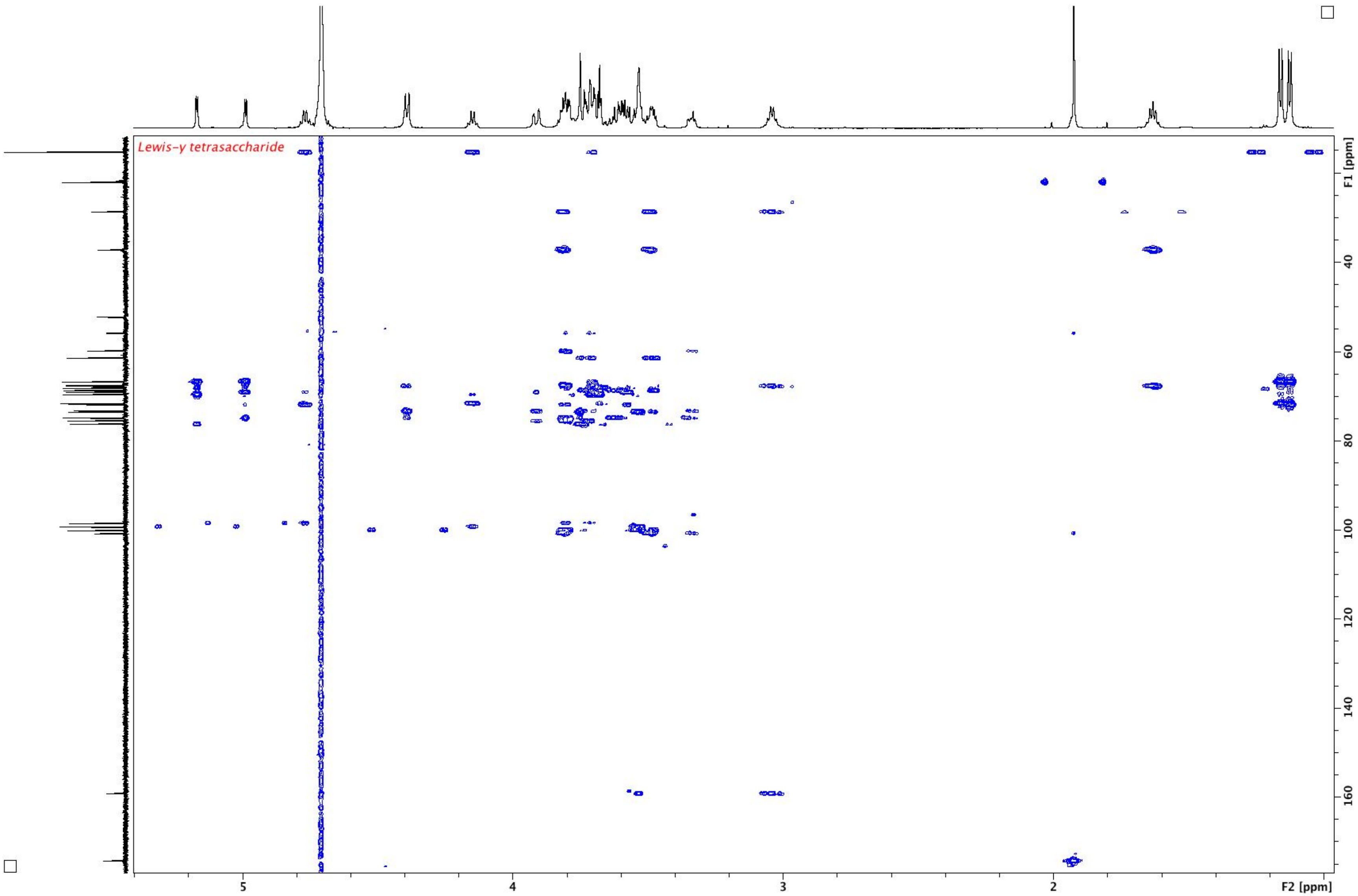

Figure S12: HMBC of Lewis-y tetrasaccharide **7**.

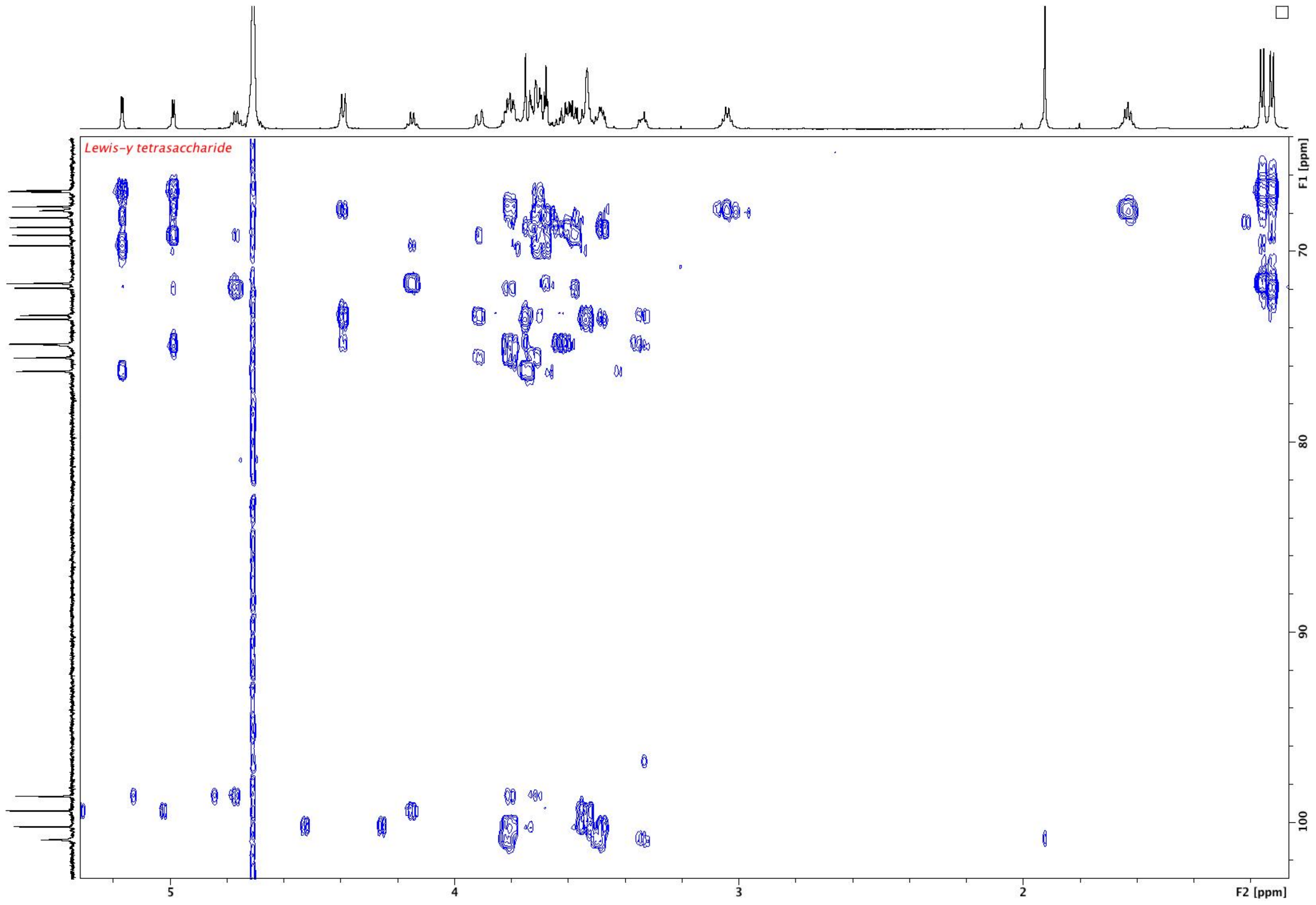

Figure S13: Expansion of the HMBC spectrum of Lewis-y tetrasaccharide **7**.

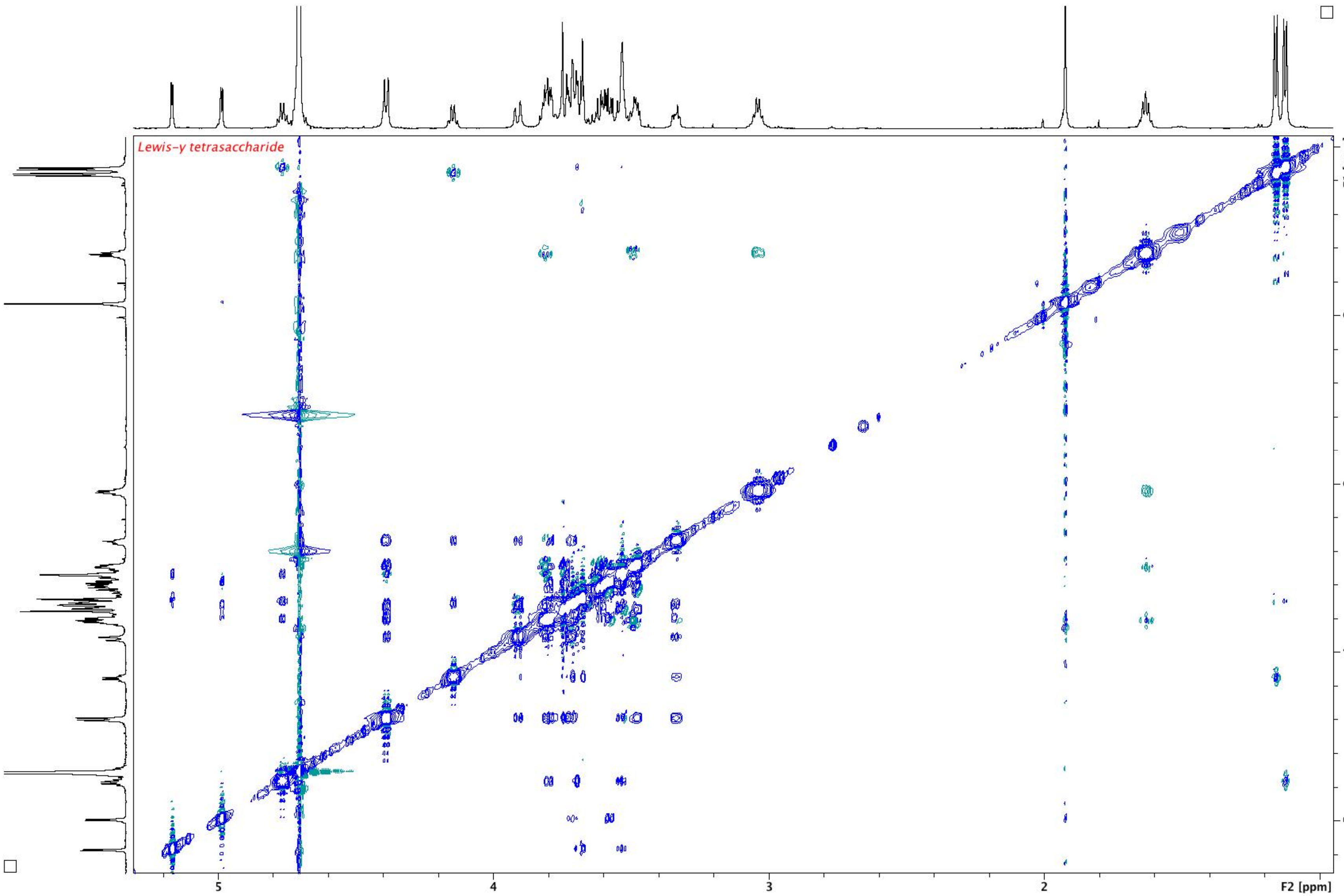

Figure S14: NOESY of Lewis-y tetrasaccharide **7**.

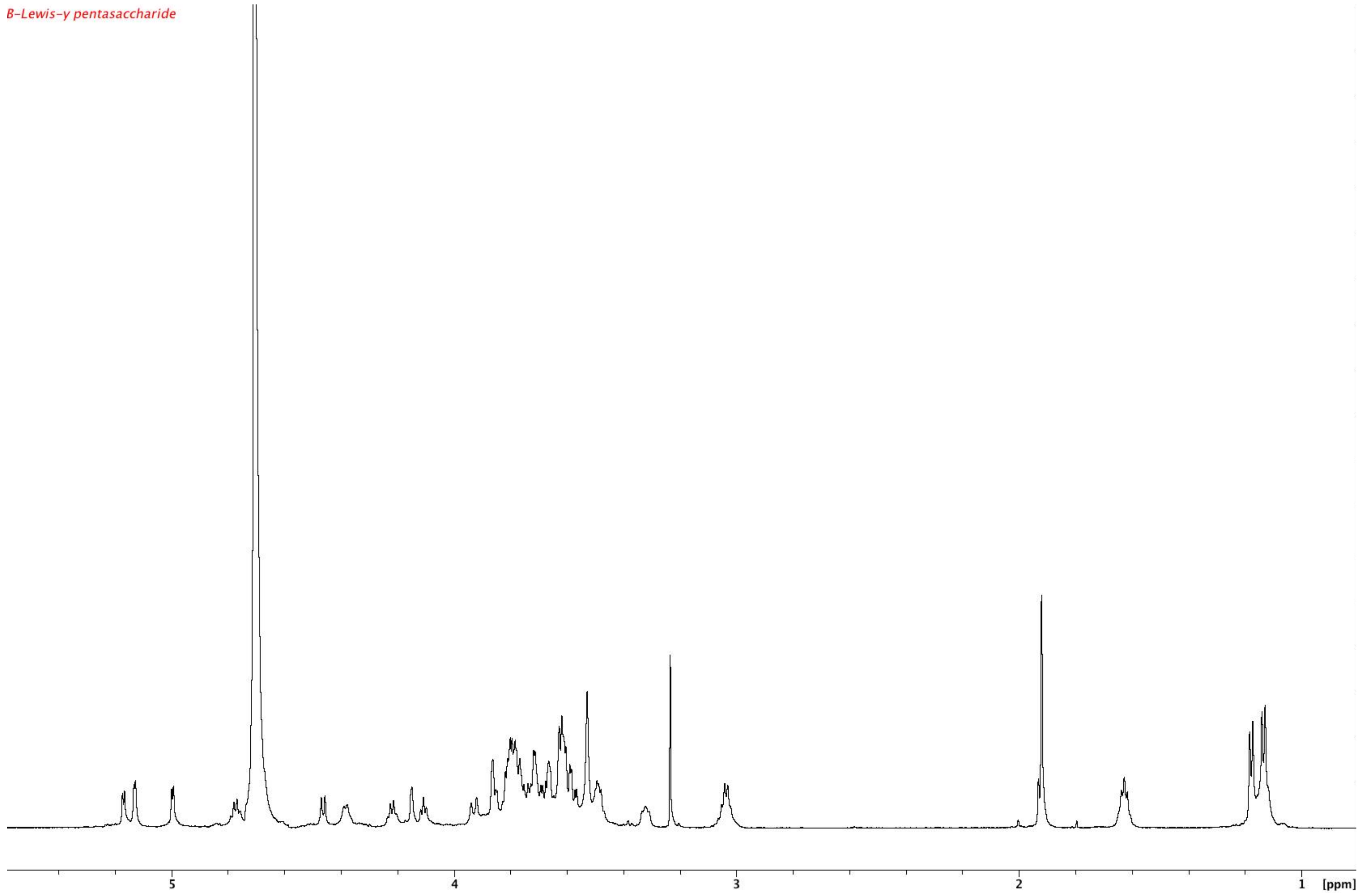

Figure S15: ^{1}H NMR spectrum of BLewis-y pentasaccharide **8**.

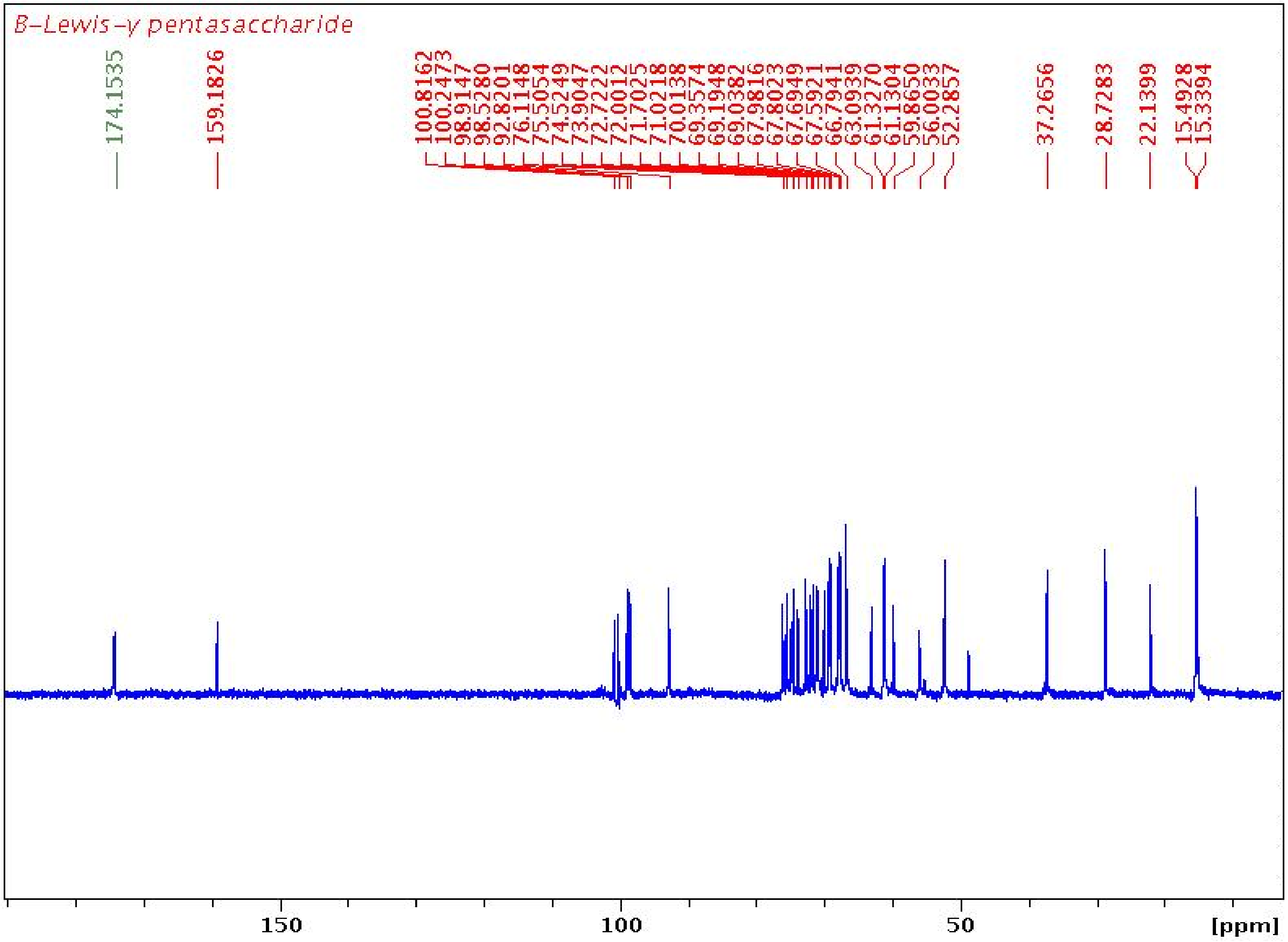

Figure S16: ^{13}C NMR spectrum of BLewis-y pentasaccharide **8**.

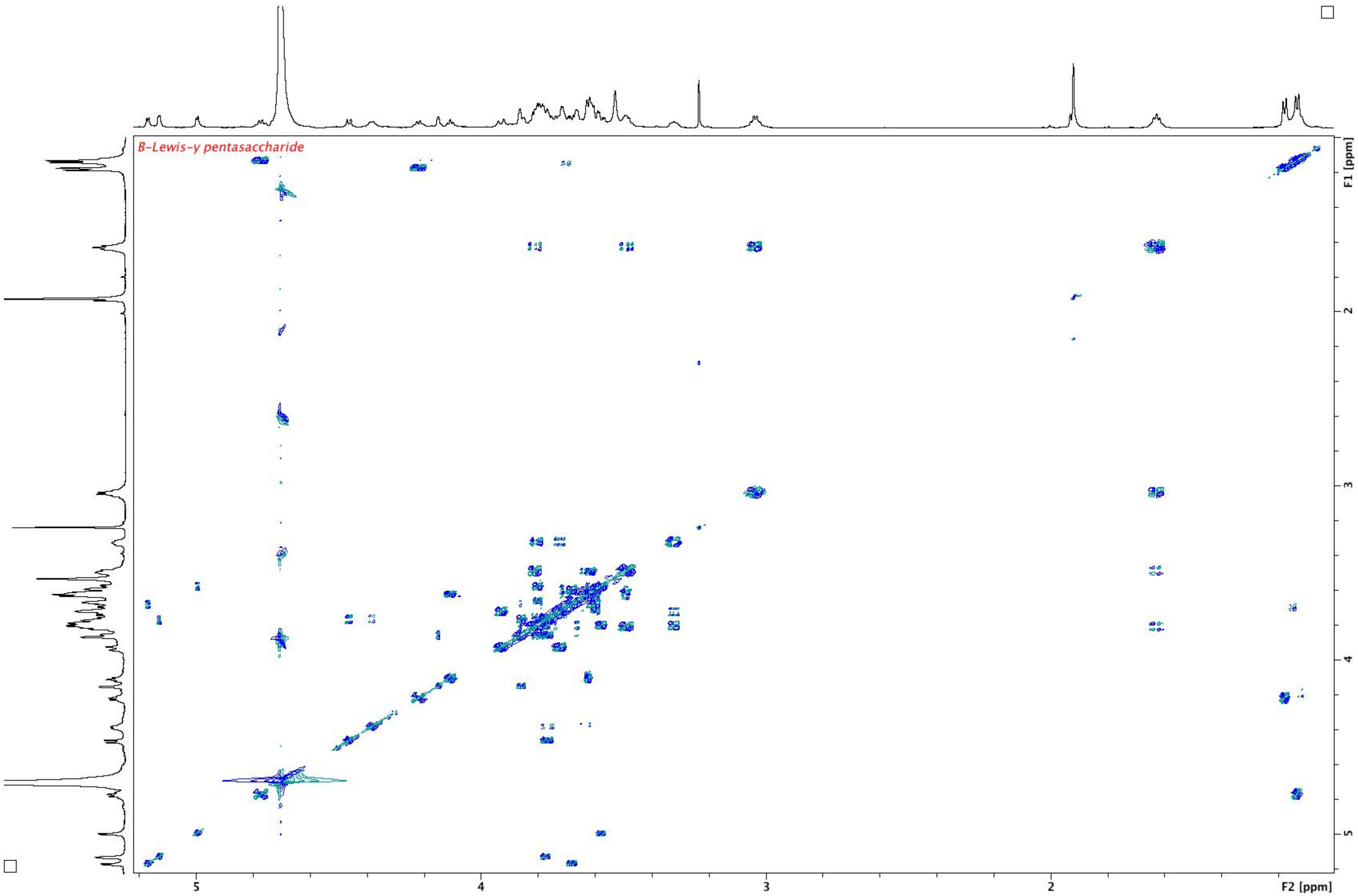

Figure S17: DQF-COSY of BLewis-y pentasaccharide **8**.

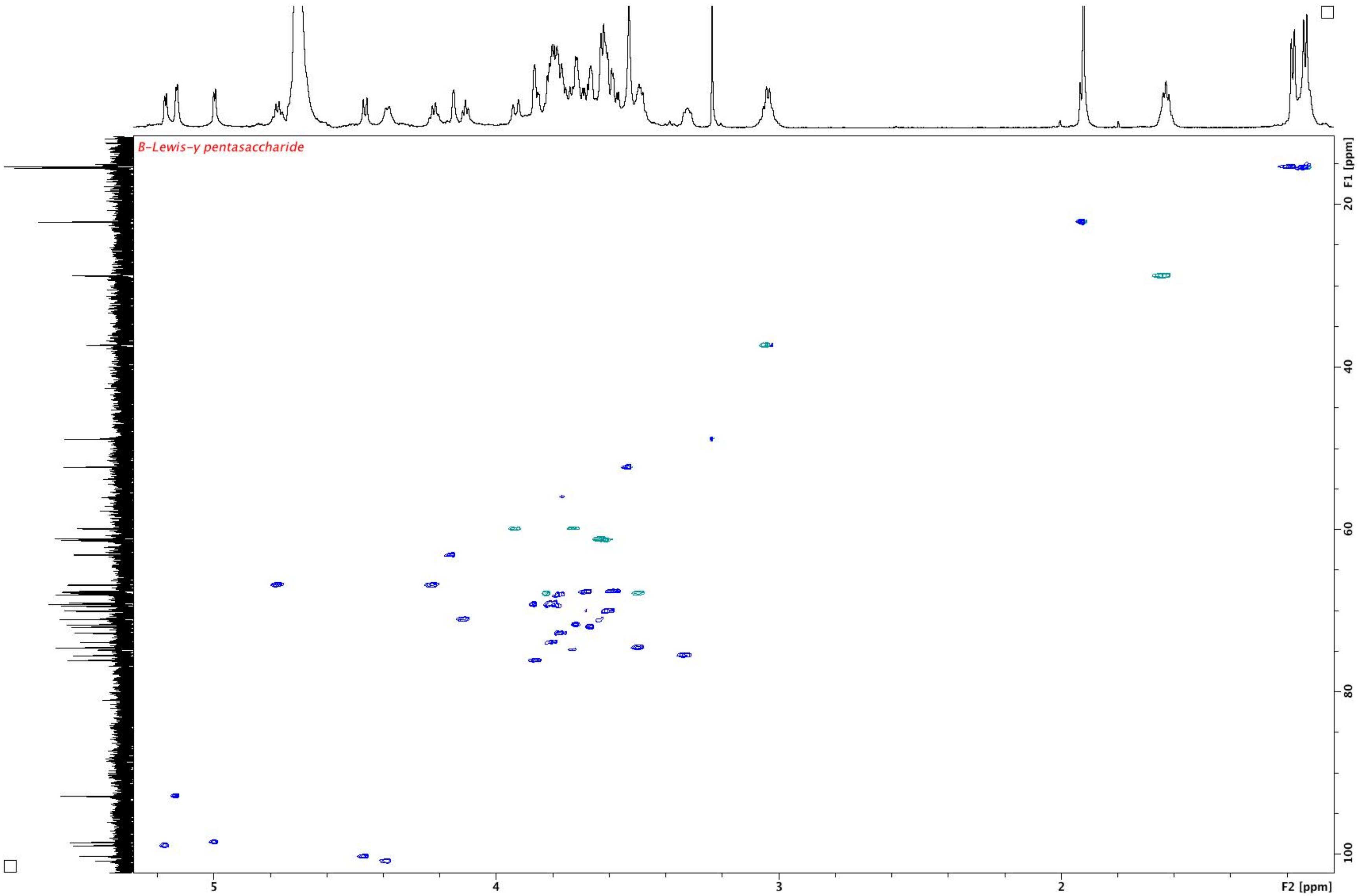

Figure S18: HSQC-edited of BLewis-y pentasaccharide **8**.

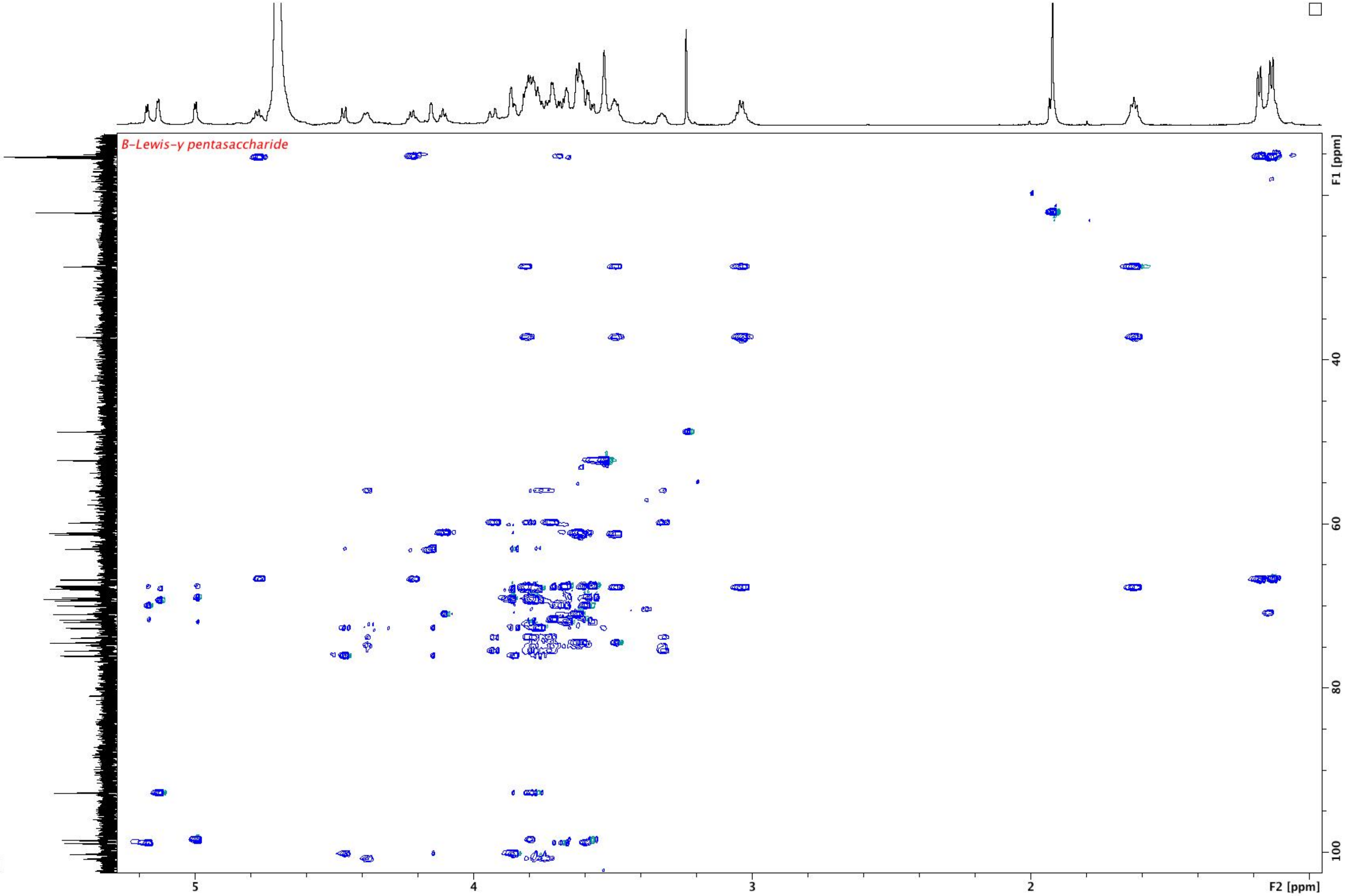

Figure S19: HSQC-TOCSY of BLewis-y pentasaccharide **8**.

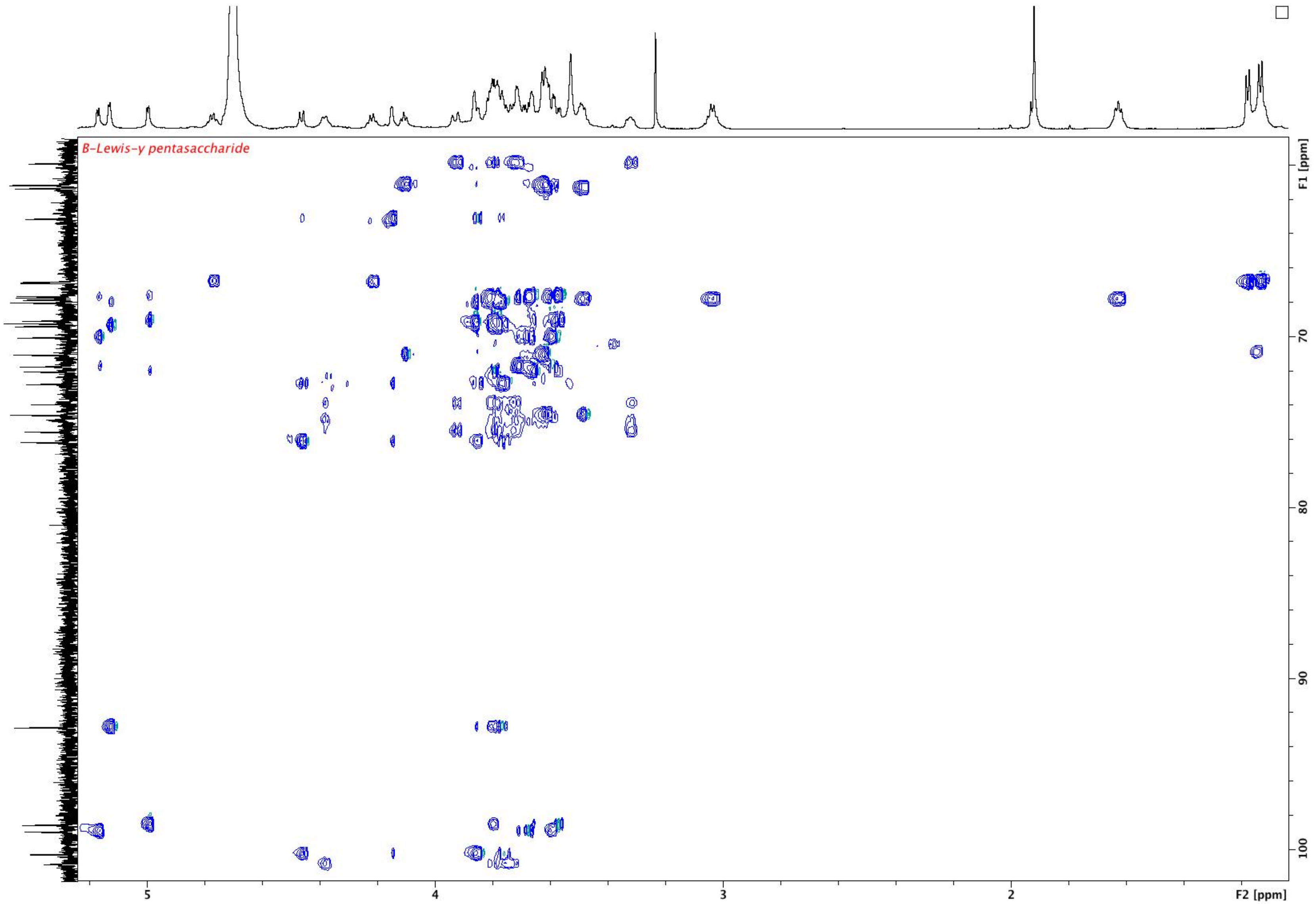

Figure S20: Expansion of the HSQC-TOCSY of BLewis-y pentasaccharide **8**.

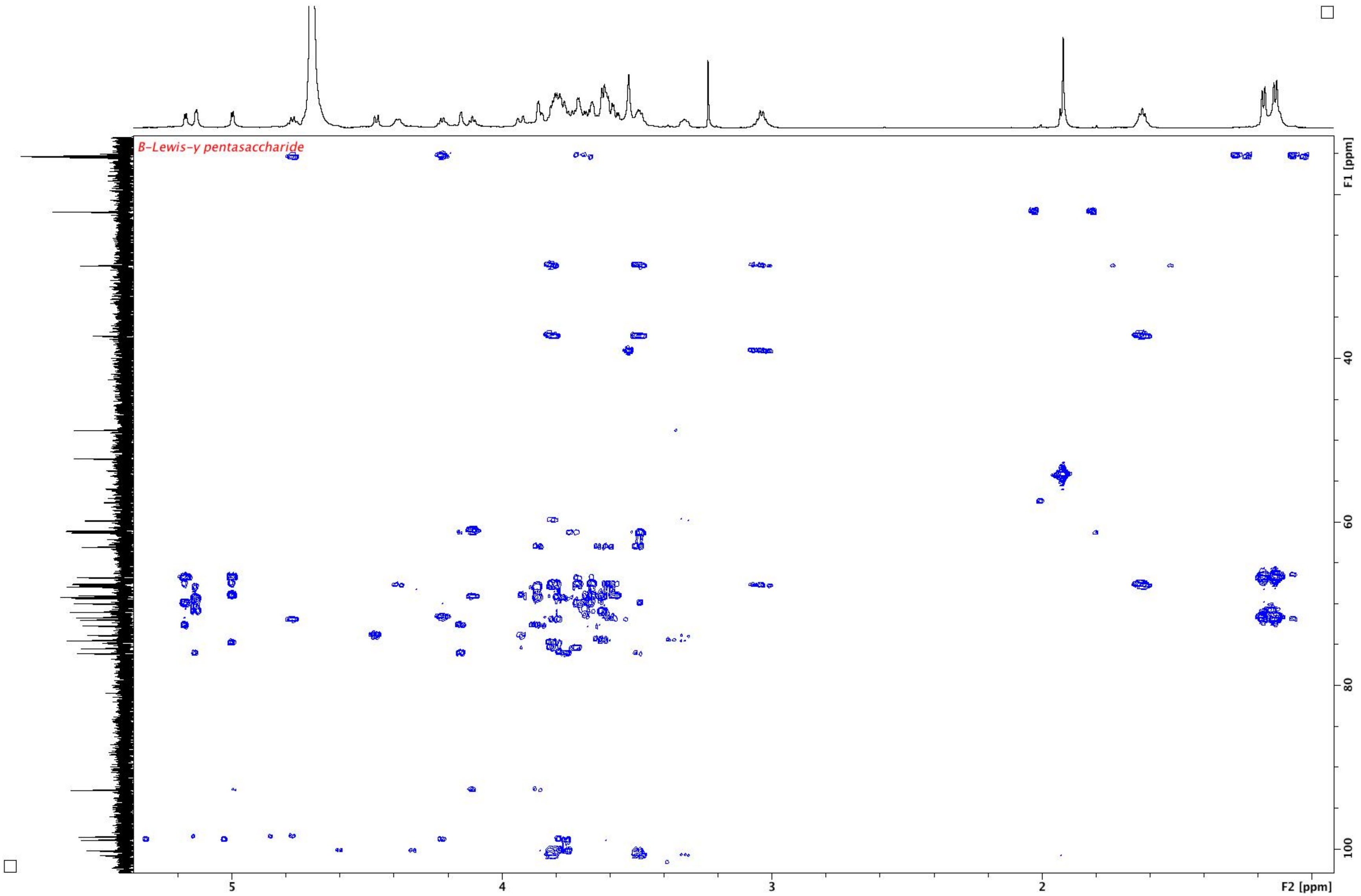

Figure S21: HMBC of BLewis-y pentasaccharide **8**.

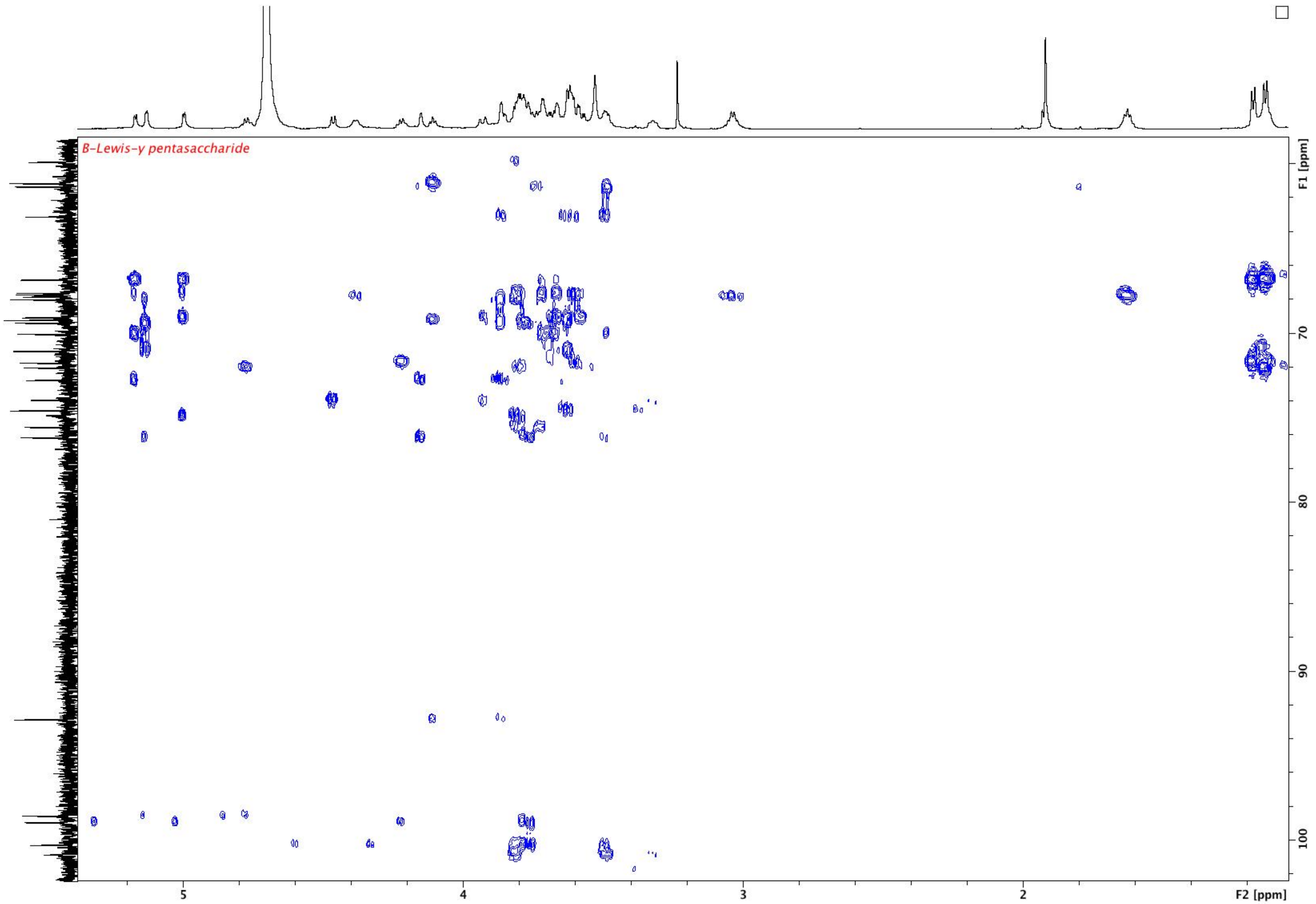

Figure S22: Expansion of the HMBC of BLewis-y pentasaccharide **8**.

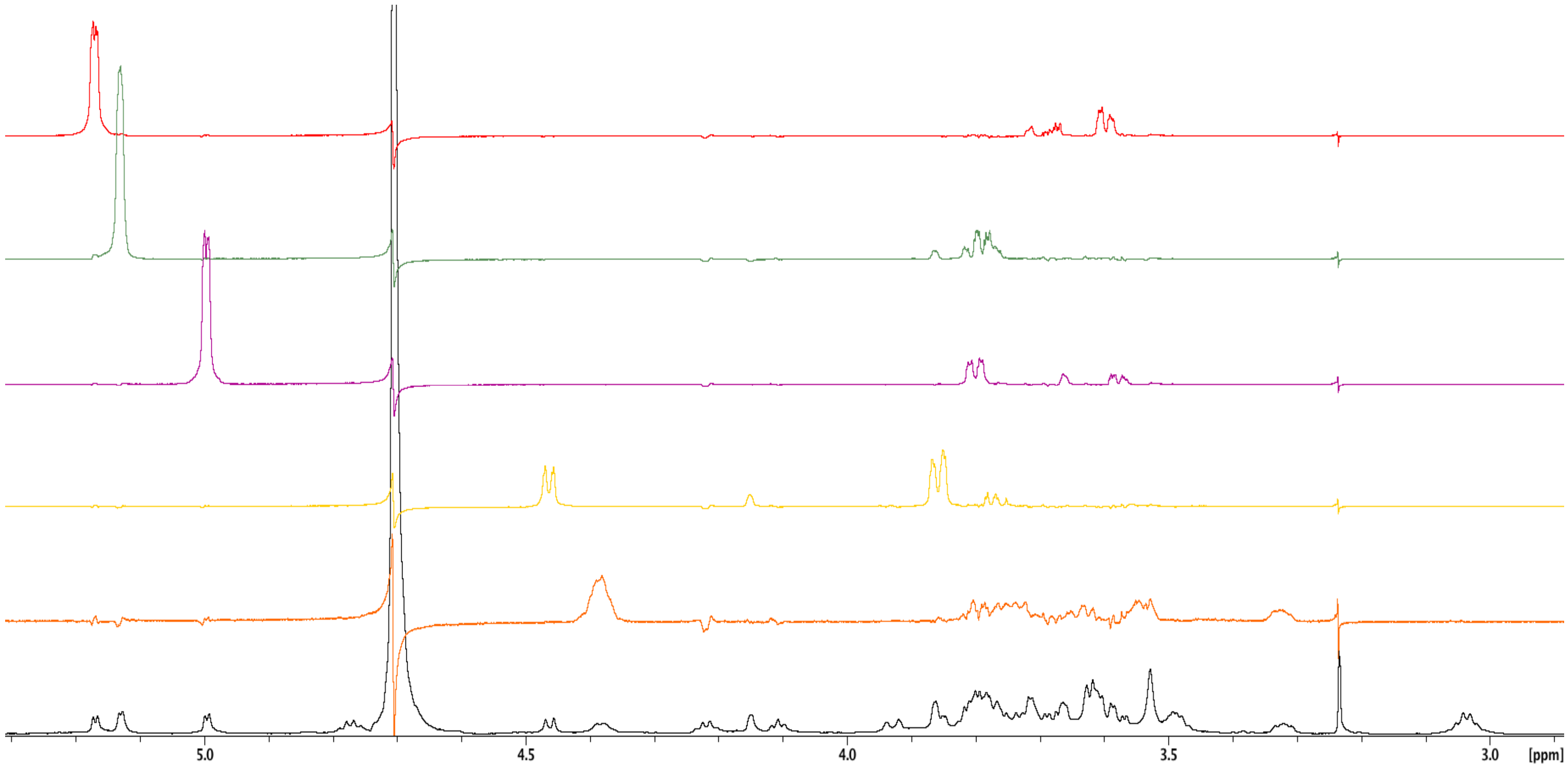

Figure S23: Stack-plot of standard ^{1}H (black) and 1D-TOCSY with selective excitation on anomeric protons of the BLewis-y pentasaccharide **8**: Fuc-D, Gal-E, Fuc-C and Gal-B and GlcNAc-A subunits from top to bottom. The TOCSY spectra were acquired with RF irradiations at 5.17 ppm (red) , 5.13 ppm (green), 4.99 ppm (purple), 4.47 ppm (yellow) and 4.40 ppm (orange), respectively.

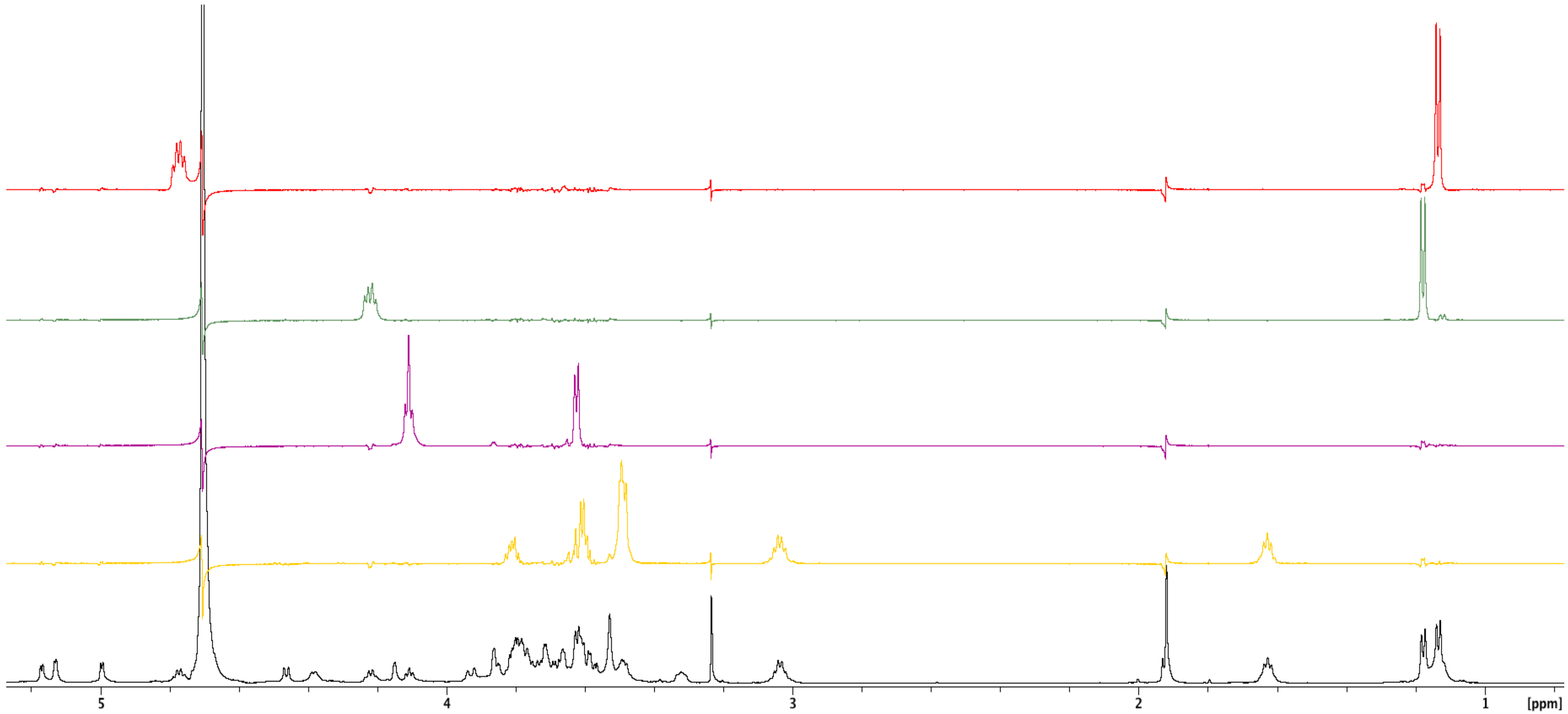

Figure S24: Stack-plot of standard ^{1}H (black) and 1D-TOCSY with selective excitation on H5 protons of the BLewis-y pentasaccharide **8**: Fuc-C, Fuc-D, Gal-E and Gal-B subunits from top to bottom (The OCH_2 protons of the side chain are overlapped with the Gal-B H5 proton). The TOCSY spectra were acquired with RF irradiations at 4.76 ppm (red) , 4.23 ppm (green), 4.10 ppm (purple) and 3.49 ppm (yellow), respectively.

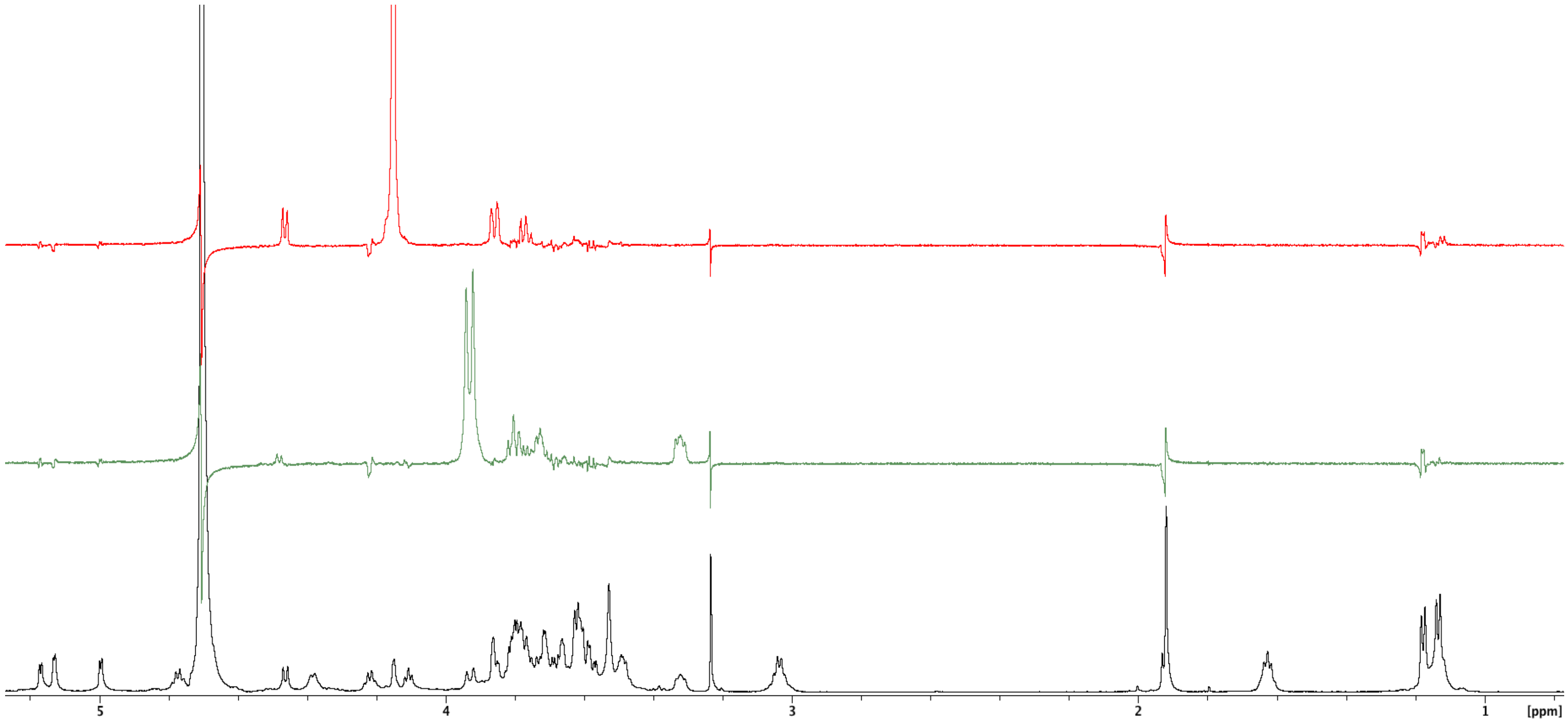

Figure S25: Stack-plot of standard ^{1}H (black) and 1D-TOCSY with selective excitation on H4-Gal-B and H6-GlcNAc-A protons of the BLewis-y pentasaccharide **8** subunits (from top to bottom). The TOCSY spectra were acquired with RF irradiations at 4.17 ppm (red) , 3.91 ppm (green), respectively.

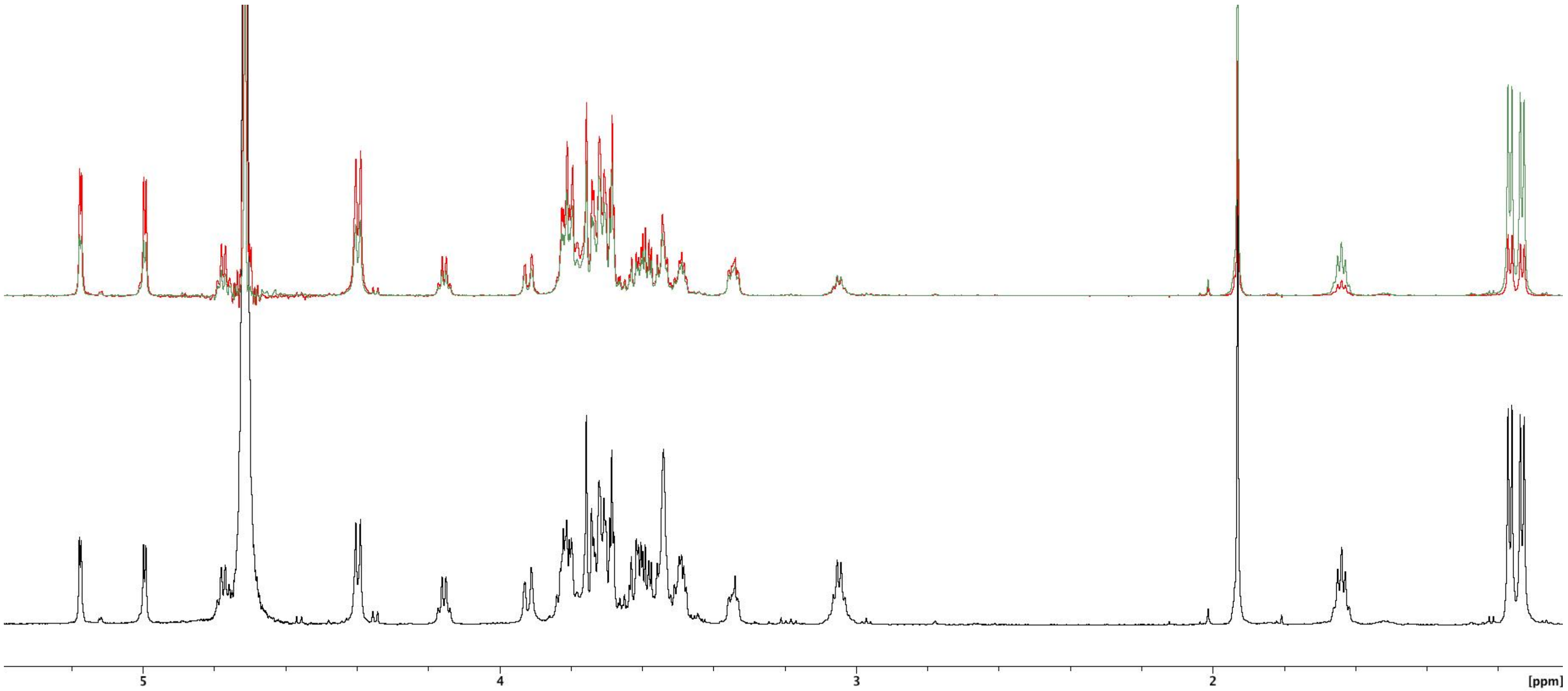

Figure S26: Standard ^{1}H (bottom) and STD-NMR spectra (top) of the CTB - Lewis-y **7** complex. The STD-NMR spectra were acquired with RF irradiations at -1 ppm (green) and 7.5 ppm (red). Receiver gain and number of scans were identical in both STD experiments.

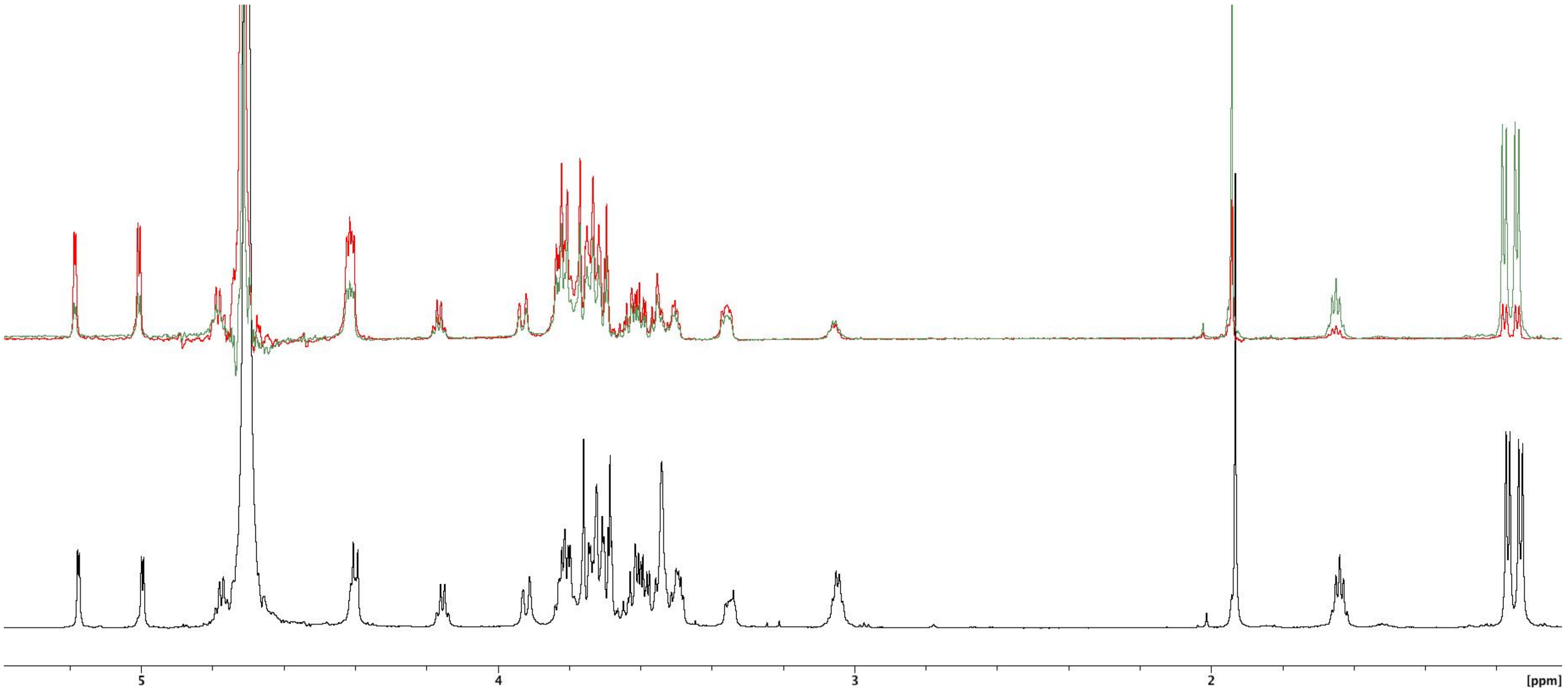

Figure S27: Standard ^{1}H (bottom) and STD-NMR spectra (top) of the LTBh - Lewis-y **7** complex. The STD-NMR spectra were acquired with RF irradiations at -1 ppm (green) and 7.5 ppm (red). Receiver gain and number of scans were identical in both STD experiments.

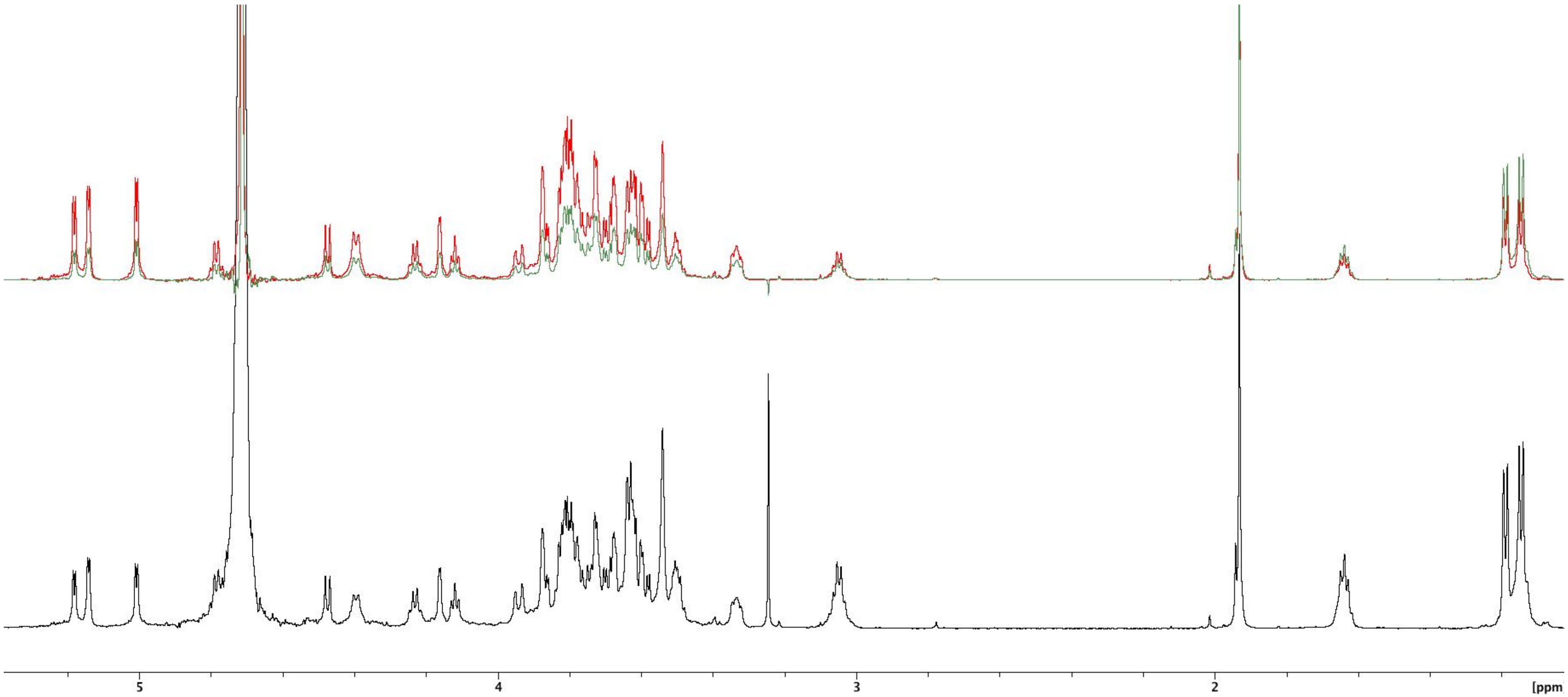

Figure S28: Standard ^{1}H (bottom) and STD-NMR spectra (top) of the LTBh - BLewis-y **8** complex. The STD-NMR spectra were acquired with RF irradiations at -1 ppm (green) and 7.5 ppm (red). Receiver gain and number of scans were identical in both STD experiments.

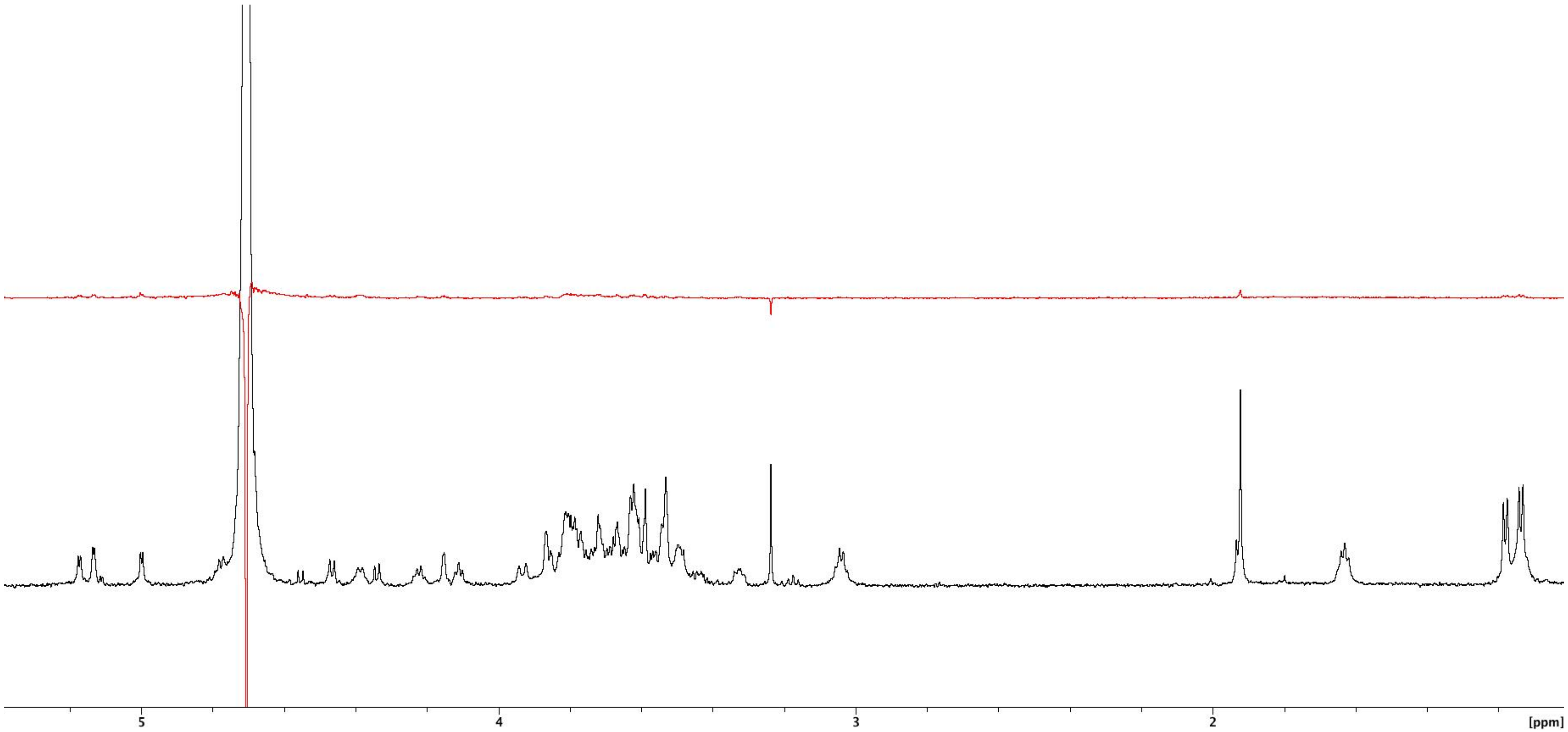

Figure S29: Standard ^{1}H (bottom) and STD-NMR spectra (top) of the CTB - BLewis-y **8** complex. The STD-NMR spectrum was acquired with a RF irradiation at 7.5 ppm (red).

Comparison of the crystal structure of LTBh with models of El Tor CTB

All CTB crystal structures in the Protein Data Bank are for classical biotype CTB with a point mutation at position 94 (His94Arg). Therefore models of El Tor CTB were constructed using the crystal structure of either classical biotype CTB (3CHB.pdb)[6] or a CTB-LTBh hybrid protein (3EFX.pdb)[8] by substitution of appropriate residues in the binding pocket. In each case three mutations were made. The models were used purely for a visual comparison with the complex of LTBh and A-Lewis-y **3b** (2O2L.pdb) in order to identify which amino acids might be responsible for preventing B-Lewis-y pentasaccharide **8** binding to El Tor CTB.[4] Ligand **8** used in our studies differs from compound **3b** in two regards: **3b** has a glucose at the reducing terminus and a GalNAc at the non-reducing terminus, whereas **8** has a GlcNAc at the reducing terminus and a Gal at the non-reducing terminus. A model for ligand **8** was constructed using the coordinates for **3b** in the 2O2L.pdb structure. The coordinates for ligand **3b** in models shown below is taken from the 3EFX.pdb structure. The relocation of the acetamide group in converting **3b** into **8** is not predicted to make any unfavourable steric clash with the proteins.

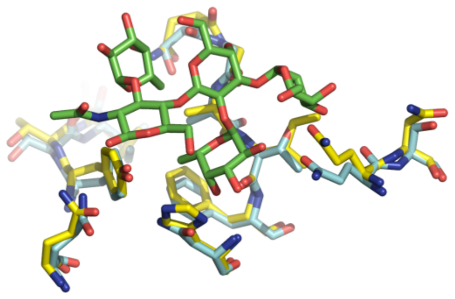

Ligand **8** (green) with El Tor CTB model derived from 3CHB.pdb (yellow) and LTBh 2O2L.pdb (blue)

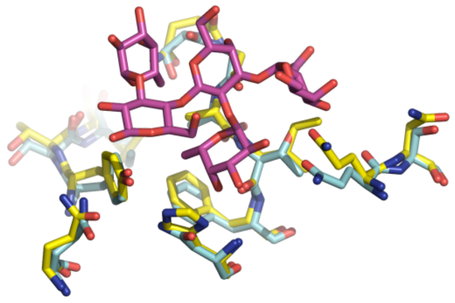

Ligand **3b** (pink) with El Tor CTB model derived from 3CHB.pdb (yellow) and LTBh 2O2L.pdb (blue)

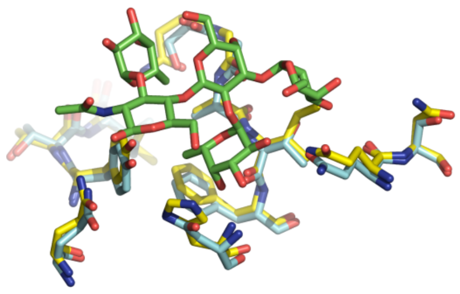

Ligand **8** (green) with El Tor CTB model derived from 3EFX.pdb (yellow) and LTBh 2O2L.pdb (blue)

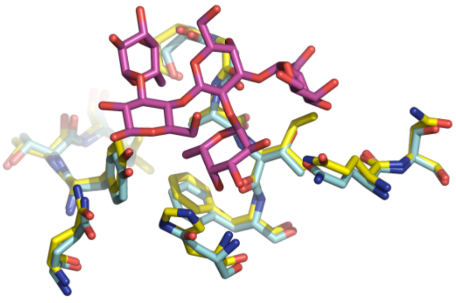

Ligand **3b** (pink) with El Tor CTB model derived from 3EFX.pdb (yellow) and LTBh 2O2L.pdb (blue)

Figure S30. Models depicting a possible binding mode for pentasaccharide **8**

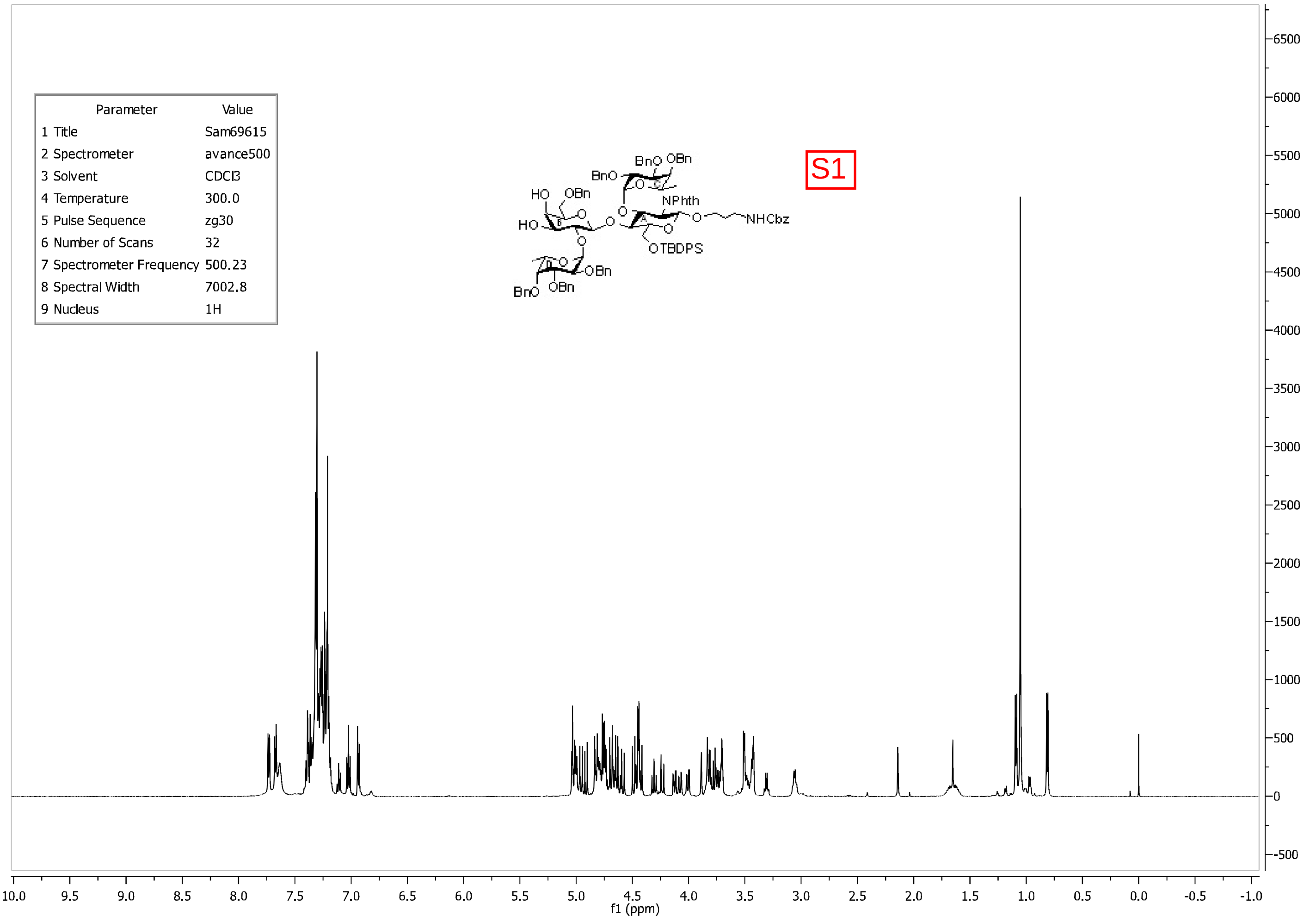

Parameter	Value
1 Title	Sam69615
2 Spectrometer	avance500
3 Solvent	CDCl3
4 Temperature	300.0
5 Pulse Sequence	zg30
6 Number of Scans	32
7 Spectrometer Frequency	500.23
8 Spectral Width	7002.8
9 Nucleus	1H

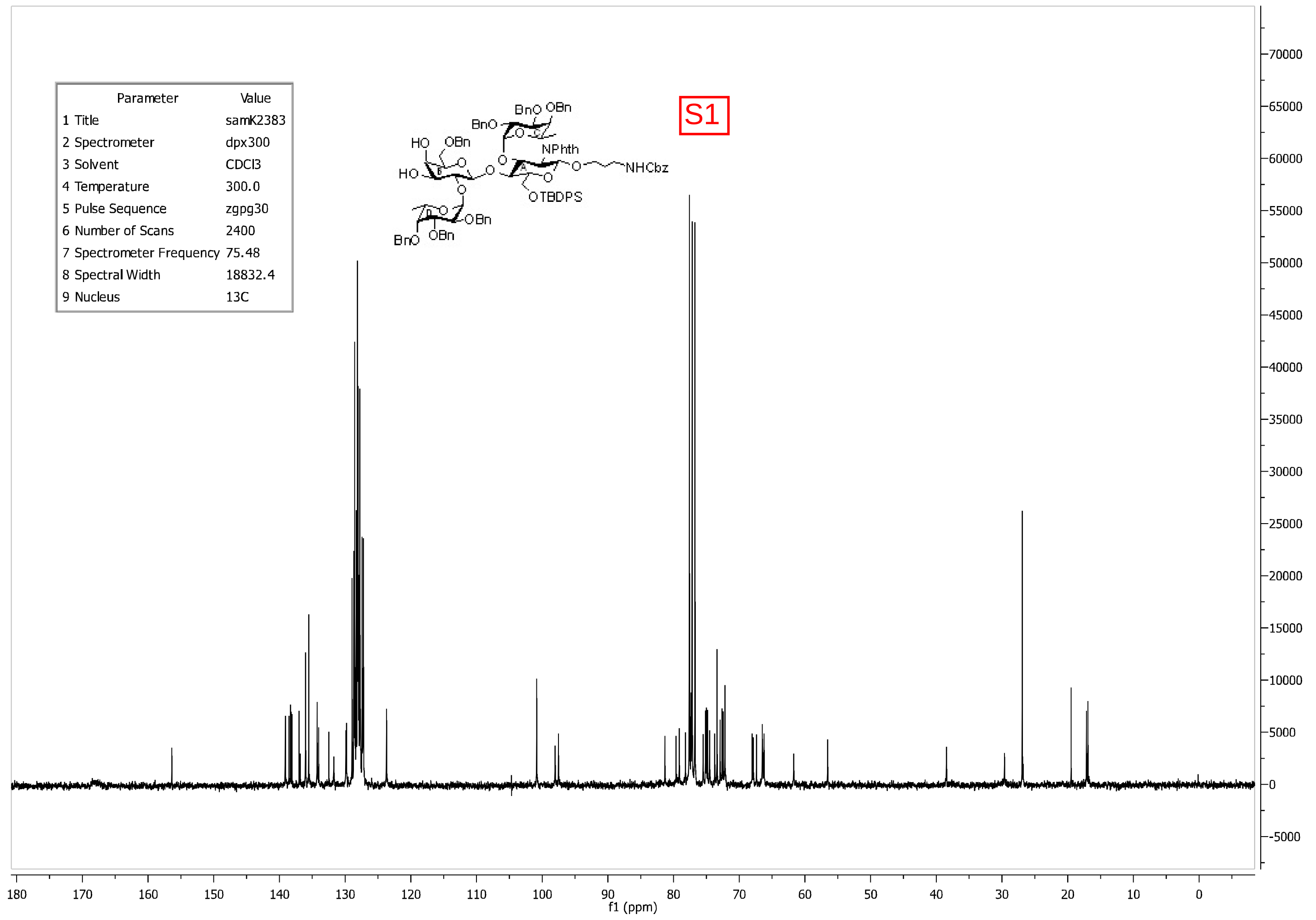

Parameter	Value
1 Title	samK2383
2 Spectrometer	dpx300
3 Solvent	CDCl3
4 Temperature	300.0
5 Pulse Sequence	zgpg30
6 Number of Scans	2400
7 Spectrometer Frequency	75.48
8 Spectral Width	18832.4
9 Nucleus	13C

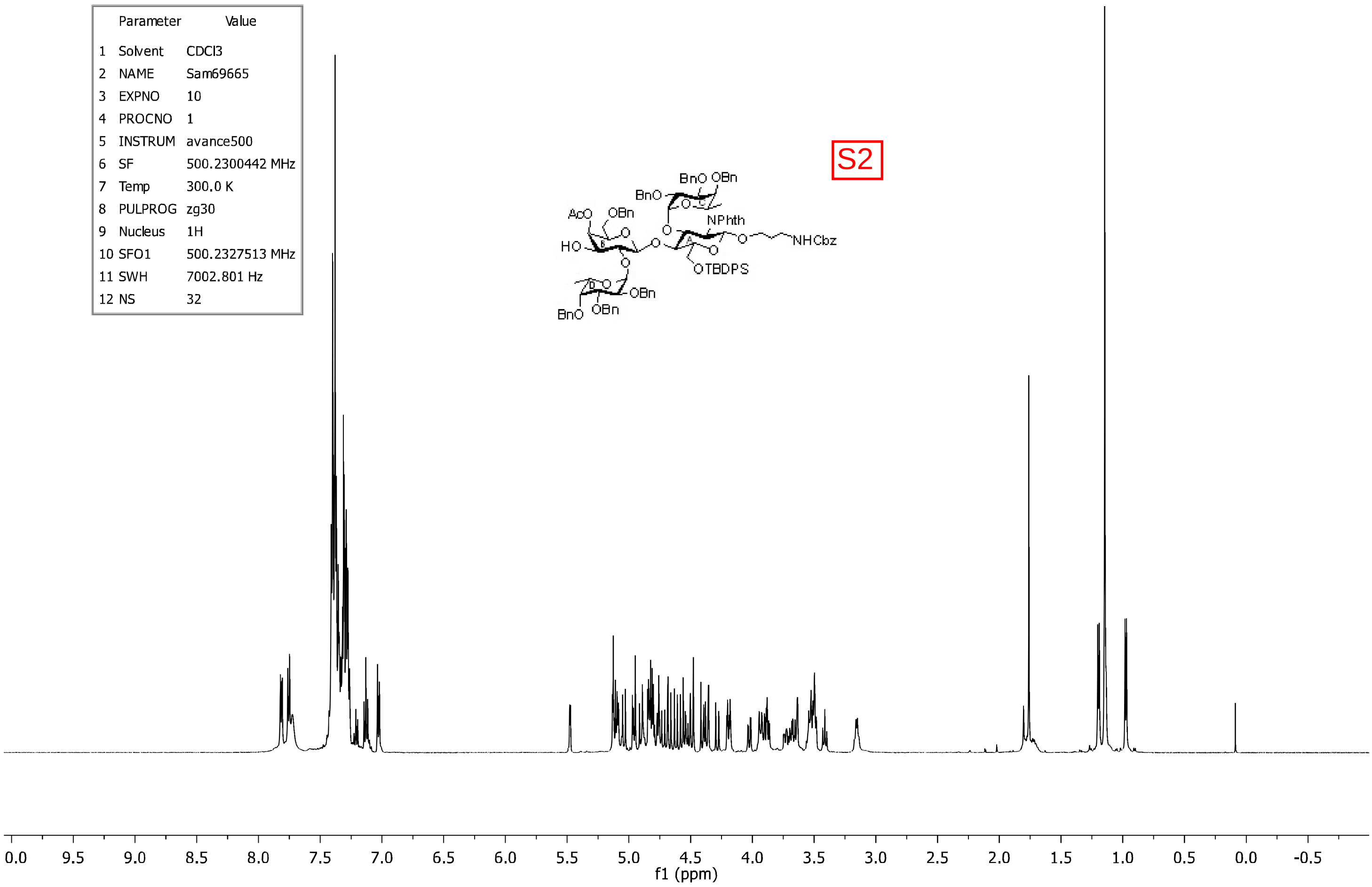
Parameter Value
1 Solvent CDCl3
2 NAME Sam69665
3 EXPNO 10
4 PROCNO 1
5 INSTRUM avance500
6 SF 500.2300442 MHz
7 Temp 300.0 K
8 PULPROG zg30
9 Nucleus 1H
10 SFO1 500.2327513 MHz
11 SWH 7002.801 Hz
12 NS 32
S2
BnO OBn
BnO
AcO OBn
NPhth
HO
NHCbz
OTBDPS
OBn
BnO OBn
f1 (ppm)

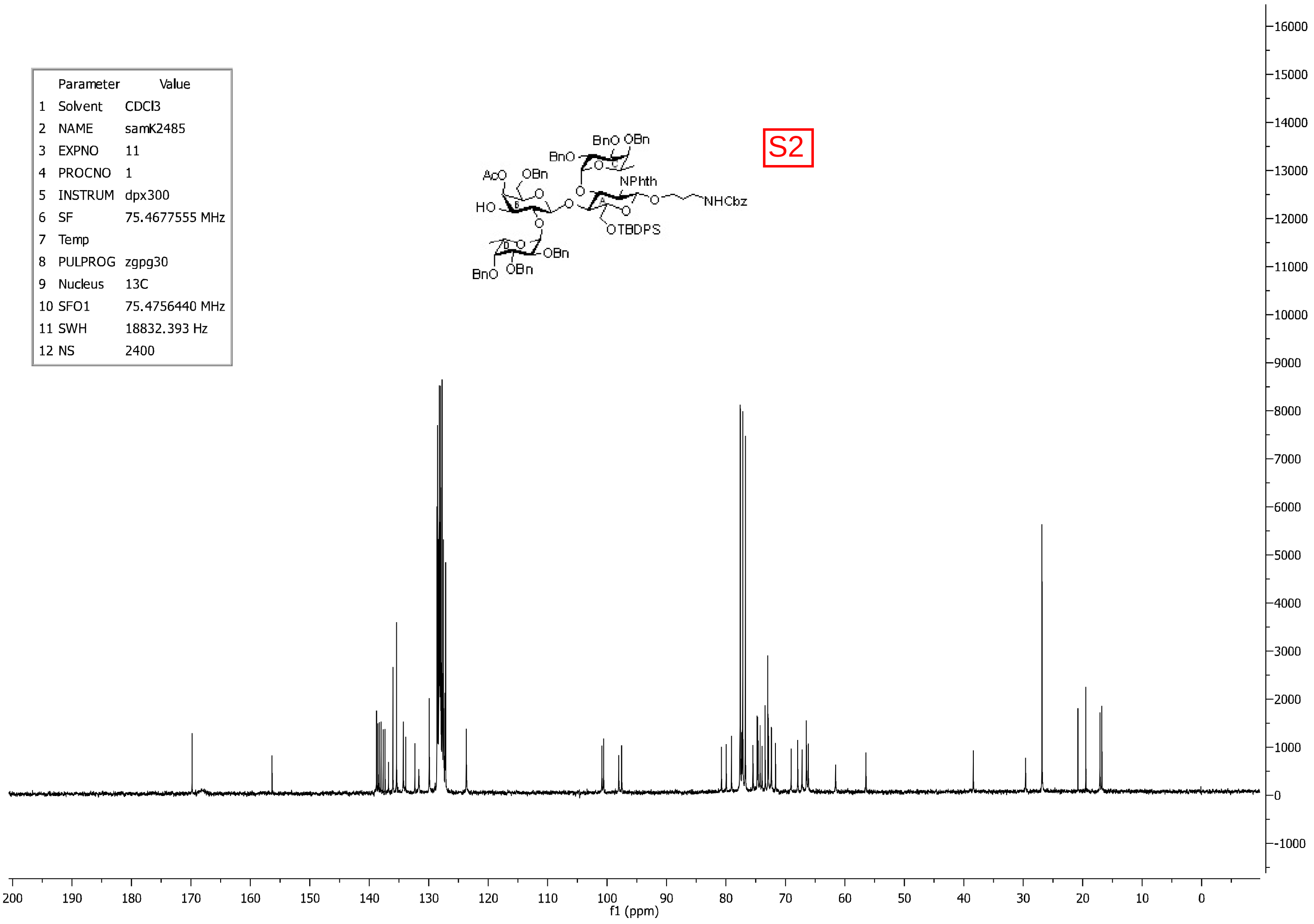
Parameter Value
1 Solvent CDCl3
2 NAME samK2485
3 EXPNO 11
4 PROCNO 1
5 INSTRUM dpx300
6 SF 75.4677555 MHz
7 Temp
8 PULPROG zgpg30
9 Nucleus 13C
10 SFO1 75.4756440 MHz
11 SWH 18832.393 Hz
12 NS 2400
S2
BnO OBn
BnO
AcO OBn
NPhth
HO
NHCbz
OTBDPS
OBn
BnO OBn
f1 (ppm)

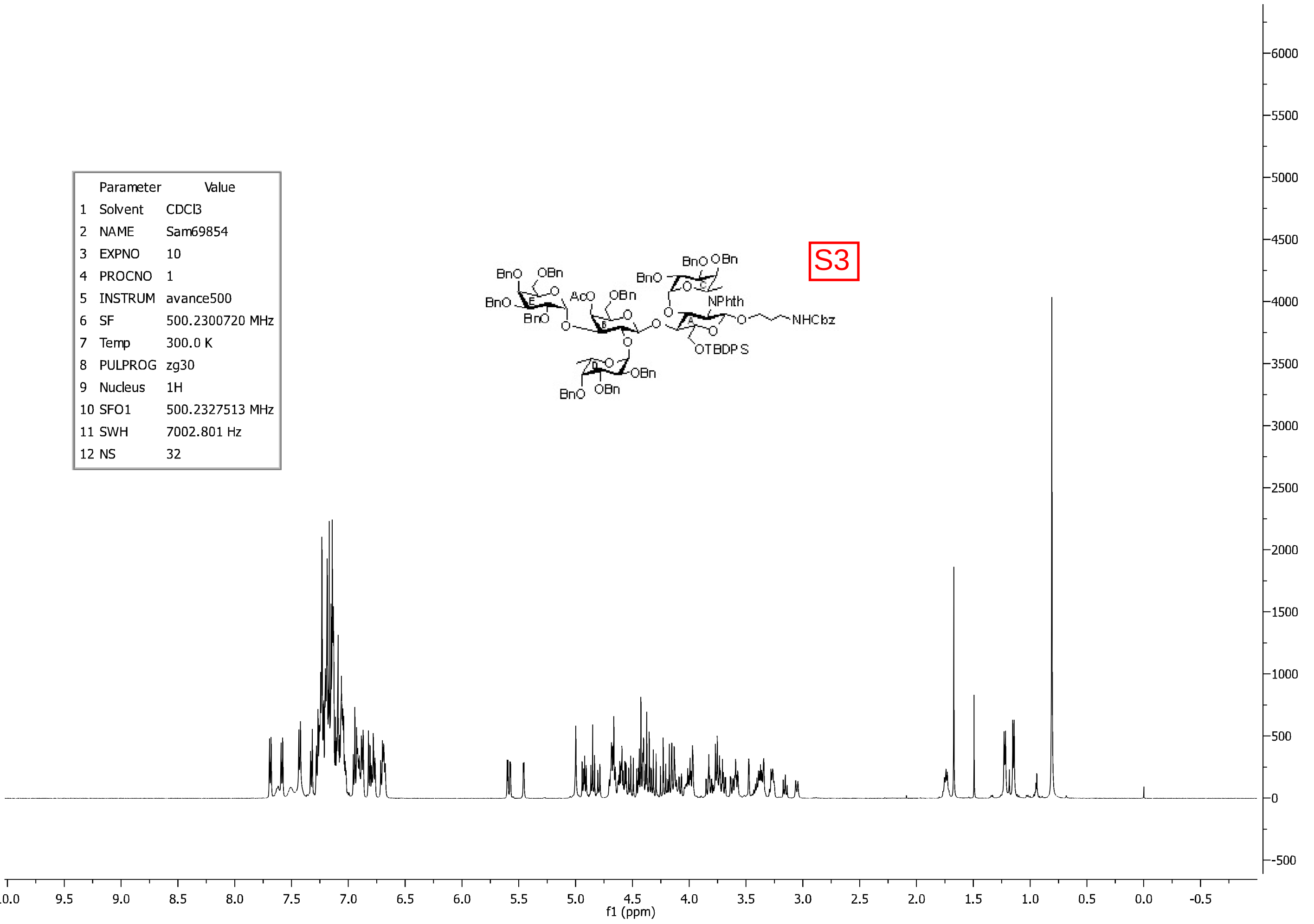

	Parameter	Value
1	Solvent	CDCl3
2	NAME	Sam69854
3	EXPNO	10
4	PROCNO	1
5	INSTRUM	avance500
6	SF	500.2300720 MHz
7	Temp	300.0 K
8	PULPROG	zg30
9	Nucleus	1H
10	SFO1	500.2327513 MHz
11	SWH	7002.801 Hz
12	NS	32

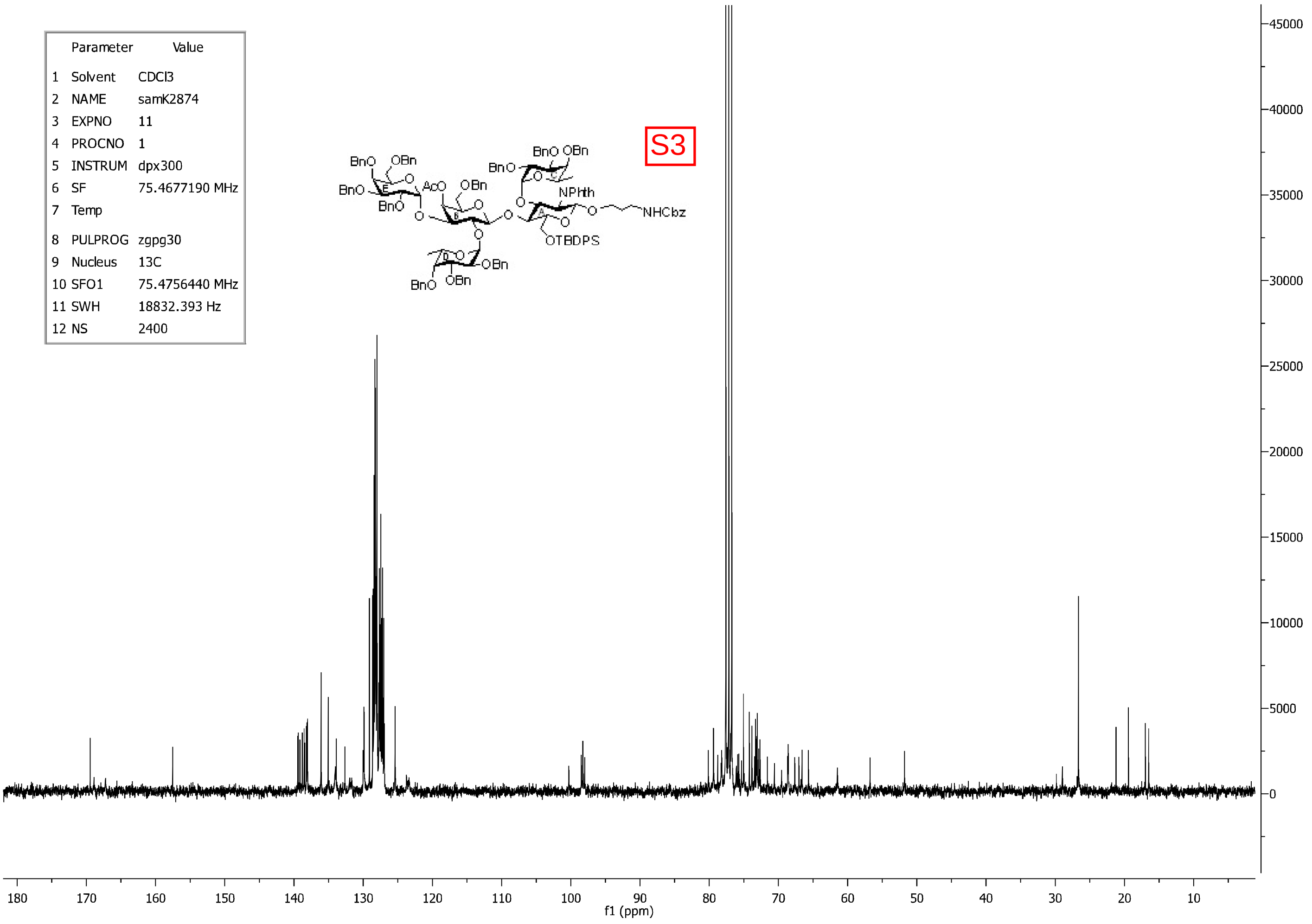

	Parameter	Value
1	Solvent	CDCl3
2	NAME	samK2874
3	EXPNO	11
4	PROCNO	1
5	INSTRUM	dpx300
6	SF	75.4677190 MHz
7	Temp	
8	PULPROG	zgpg30
9	Nucleus	13C
10	SFO1	75.4756440 MHz
11	SWH	18832.393 Hz
12	NS	2400

References

[1] P. K. Mandal, W. B. Turnbull, *Carbohydr. Res.* **2011**, *346*, 2113-2120.

[2] M. Sandkvist, T. R. Hirst, M. Bagdasarian, *J. Bacteriol.* **1987**, *169*, 4570-4576.

[3] A. T. Aman, S. Fraser, E. A. Merritt, C. Rodigherio, M. Kenny, M. Ahn, W. G. J. Hol, N. A. Williams, W. I. Lencer, T. R. Hirst, *Proc. Natl. Acad. Sci. USA* **2001**, *98*, 8536-8541.

[4] A. Holmner, G. Askarieh, M. Ökvist, U. Krengel, *J. Mol. Biol.* **2007**, *371*, 754-764.

[5] a) O. Olsvik, J. Wahlberg, B. Petterson, M. Uhlen, T. Popovic, I. K. Wachsmuth, P. I. Fields, *J. Clin. Microbiol.* **1993**, *31*, 22-25; b) M. Lebens, J. Holmgren, *FEMS Microbiol. Lett.* **1994**, *117*, 197-202.

[6] E. A. Merritt, P. Kuhn, S. Sarfaty, J. L. Erbe, R. K. Holmes, W. G. J. Hol, *J. Mol. Biol.* **1998**, *282*, 1043-1059.

[7] W. B. Turnbull, A. H. Daranas, *J. Am. Chem. Soc.* **2003**, *125*, 14859-14866.

[8] A. Holmner, M. Lebens, S. Teneberg, J. Angstrom, M. Ökvist, U. Krengel, *Structure* **2004**, *12*, 1655-1667.
